# Supplementary material for: The reactive pyruvate metabolite dimethylglyoxal mediates neurological consequences of diabetes
Source: Nat Commun. 2024 Jul 10;15:5745. doi: 10.1038/s41467-024-50089-3 (PMC11237006; doi:10.1038/s41467-024-50089-3)
Supplement: Supplementary file 1 — Supplementary Information [file 41467_2024_50089_MOESM1_ESM.pdf]

Supplementary Information

Supplementary Fig. 1

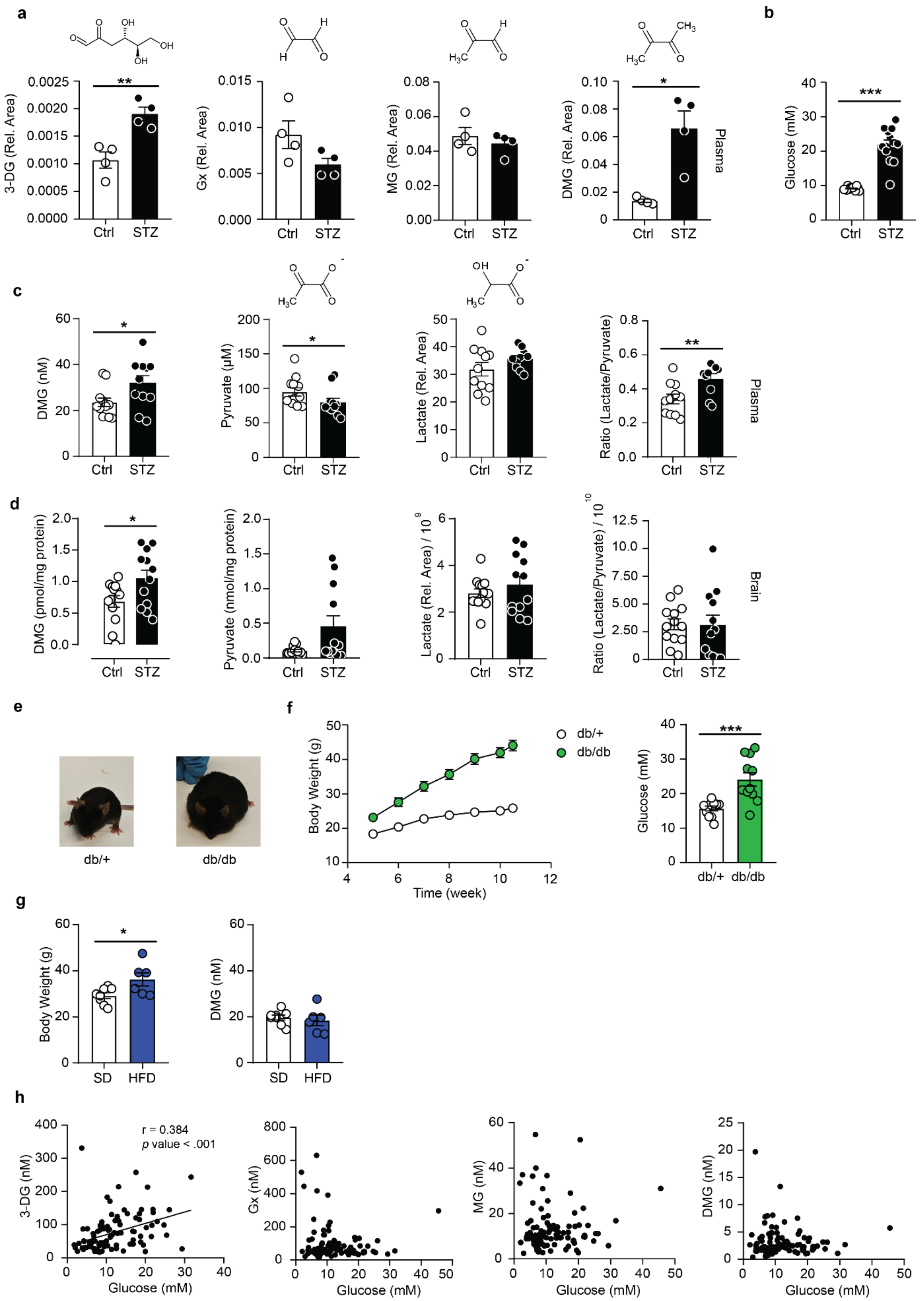

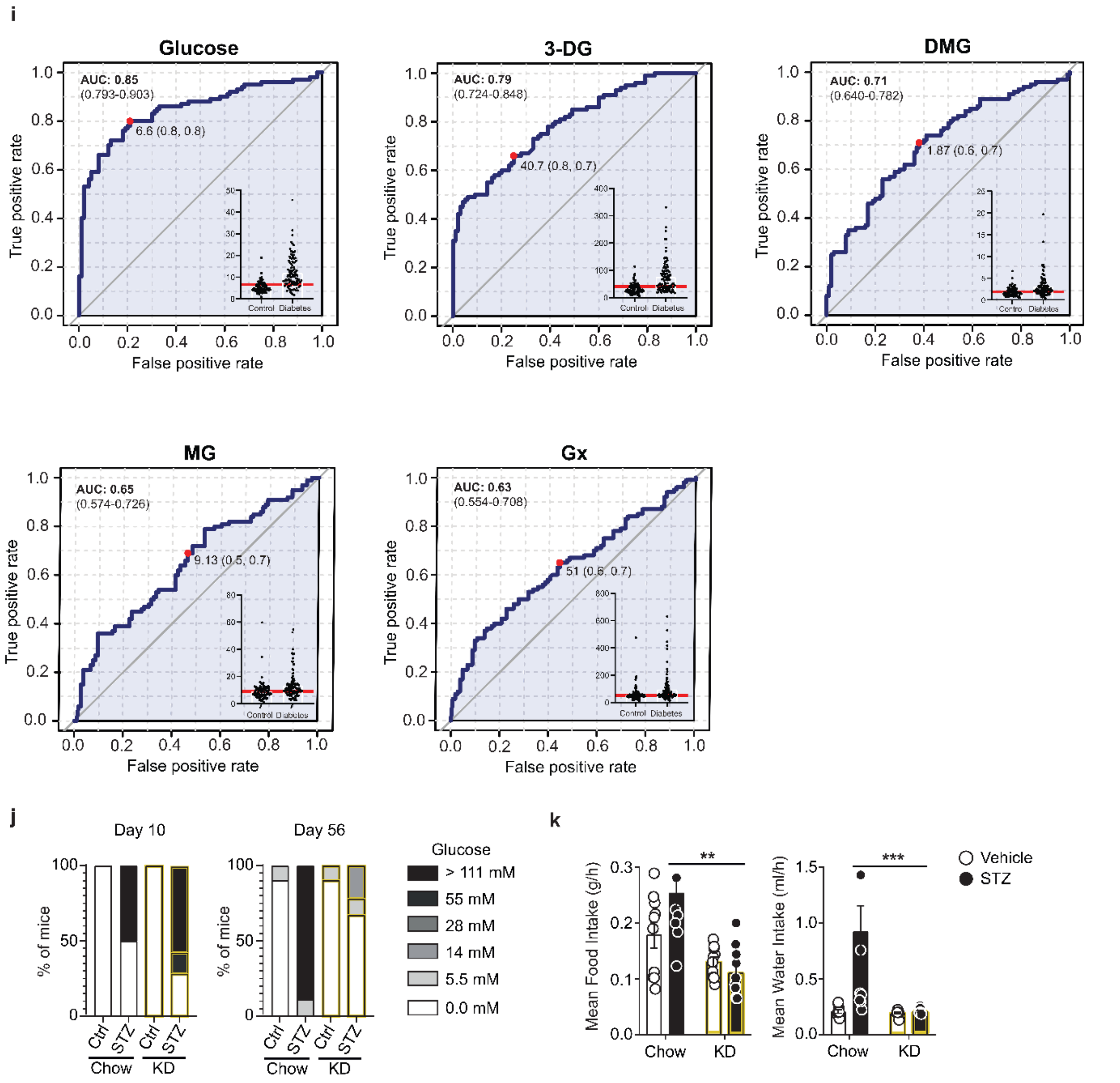

Supplementary Fig. 1 | Glucose and α-dicarbonyl levels in experimental and human diabetes and the influence of ketogenic diet. a Concentrations of α-dicarbonyls in plasma 1 week after streptozotocin (STZ; *n*=4 per group) injections (Fig. 1a). **b** Blood glucose concentrations in the second STZ-treated cohort of mice 15 weeks after inducing diabetes (*n*=13 per group). **c** Dimethylglyoxal (DMG), pyruvate and lactate levels and lactate-pyruvate ratio in plasma 15 weeks after STZ (*n*=10-11) or control (*n*=11-12) injections. **d** DMG, pyruvate and lactate levels and lactate-pyruvate ratio in brain 15 weeks after STZ (*n*=12) or control (*n*=12-13) injections. **e** Pictures of db/+ mouse and db/db mouse. **f** Body weight over time (*n*=12 per group) and blood glucose concentrations (*n*=11 per group) in 10-11-week-old db/+ and db/db mice. **g** Body weight and serum DMG concentrations of mice fed for 12 weeks with standard diet (SD; *n*=8) or high fat diet (HFD; *n*=6). **h** Correlation between α-dicarbonyl and glucose concentrations in serum of patients with diabetes (*n*=98-99). **i** Univariate receiver operating characteristic (ROC) curve analysis for glucose and α-dicarbonyls to assess the diagnostic performance for patients with diabetes (*n*=98-100). Red dot and line indicate the best cut-off/decision threshold with specificity and sensitivity in parenthesis. Area under the curve (AUC) with 95% confidence interval (CI) in parenthesis. Analysis was performed using MetaboAnalyst 5.0. **j** Glucose concentrations in urine 10 and 56 days after mice received vehicle or STZ (Fig. 3a). **k** Food and water intake of mice (*n*=10 per group). Values are means ± SEM. Statistical comparison by two-tailed unpaired t test (a (3-DG, Gx and MG), c (DMG and lactate-pyruvate ratio), d (DMG) and g), two-tailed Mann-Whitney U test (a (DMG), b, c (pyruvate and lactate), d (pyruvate, lactate and lactate-pyruvate ratio) and f (glucose)), 2-way repeated measures ANOVA (f (body weight – time)), Pearson correlation coefficient (h), receiver operating characteristic (ROC) curve (i) or Scheirer-Ray-Hare test followed by

two-tailed Mann-Whitney U post hoc tests and Bonferroni-Holm correction (k). **p* < .05; ***p* < .01; ****p* < .001. Detailed information on the test statistics is provided in Supplementary Table 6.

Supplementary Fig. 2

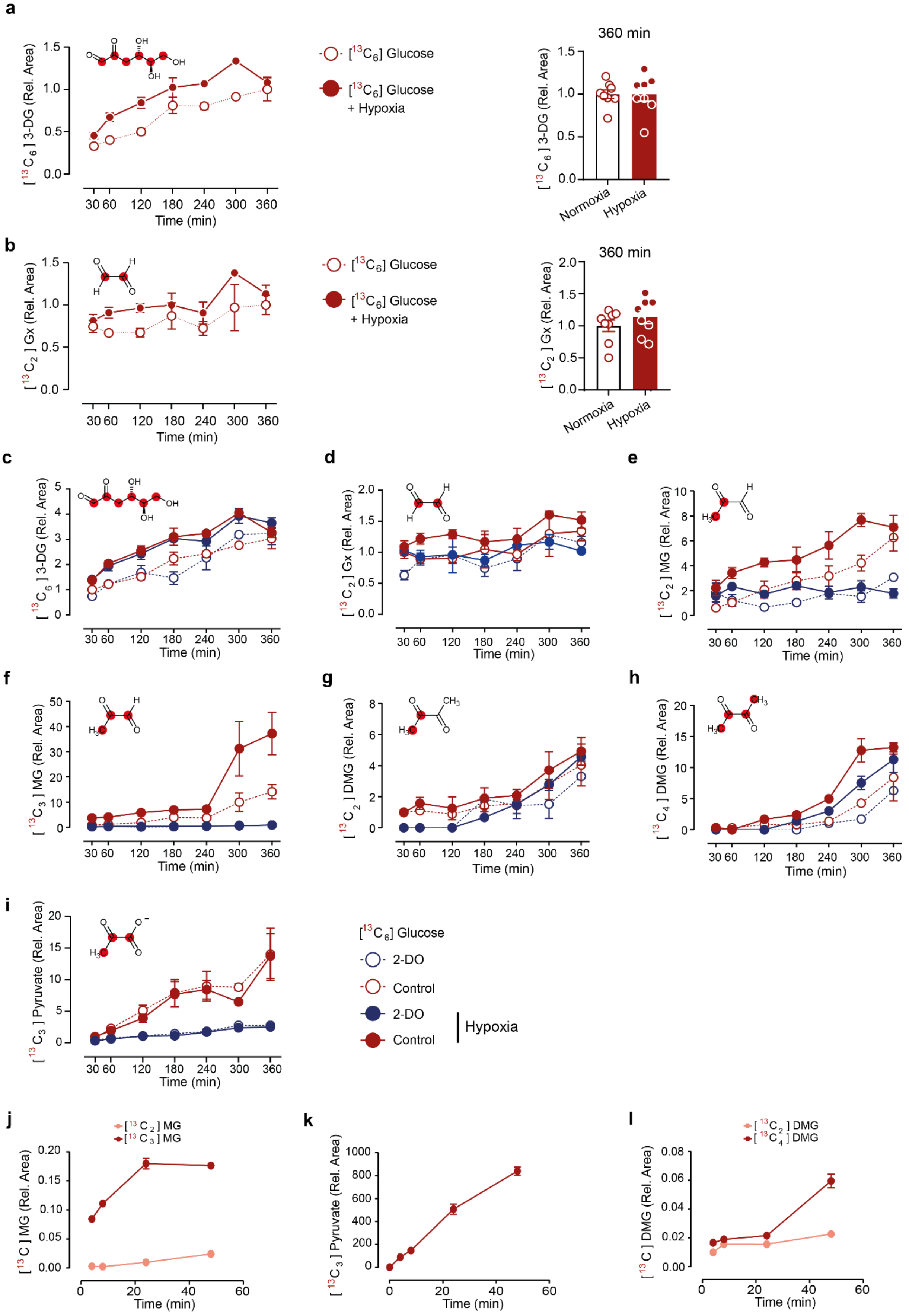

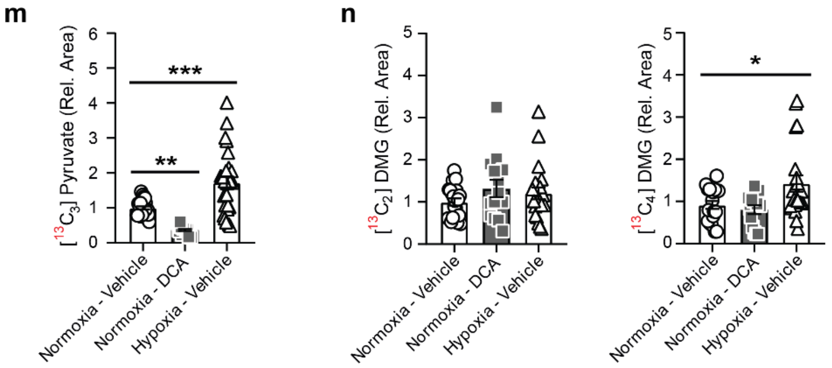

Supplementary Fig. 2 | Dimethylglyoxal is a distant glucose metabolite. a, b Left panels, time course of [$^{13}C_6$]-3-deoxyglucosone ([$^{13}C_6$]3-DG) and [$^{13}C_2$]-glyoxal ([$^{13}C_2$]Gx) in medium after adding [$^{13}C_6$]-glucose to bEnd.3 cells and changing to hypoxic conditions as indicated (descriptive only). Right panels, fully ^{13}C-labeled 3-DG and Gx under normoxic or hypoxic conditions at time point 360 min in an independent experiment (*n*=8 per group). Dicarbonyl levels were normalized to the parallel 360-min normoxia group. **c-i** Effects of 2-deoxyglucose (2-DO) on [^{13}C]-dicarbonyls and [$^{13}C_3$]-pyruvate generation under normoxic or hypoxic conditions (descriptive only). **j-l** [^{13}C]-methylglyoxal ([^{13}C]MG), [$^{13}C_3$]-pyruvate, and [^{13}C]-dimethylglyoxal ([^{13}C]DMG) in medium after treatment of mouse brain endothelial (bEnd.3) cells with [$^{13}C_6$]-glucose for 50 minutes (descriptive only). **m** [$^{13}C_3$]-pyruvate in medium 6 hours after treatment with [$^{13}C_6$]-glucose and 30 mM dichloroacetate (DCA) under normoxic (*n*=25 (vehicle), *n*=17 (DCA)) or hypoxic conditions (*n*=26). **n** [$^{13}C_2$]DMG, and [$^{13}C_4$]DMG in medium 6 hours after treatment with [$^{13}C_6$]-glucose and 30 mM dichloroacetate (DCA) under normoxic (*n*=19-20 (vehicle), *n*=14 (DCA)) or hypoxic conditions (*n*=21-22). Values are means ± SEM. Statistical comparison by two-tailed unpaired t test (a and b) or one-way ANOVA followed by Dunnett´s post-hoc tests (m and n) **p* < .05; ***p* < .01; ****p* < .001. Detailed information on the test statistics is provided in Supplementary Table 6.

Supplementary Fig. 3

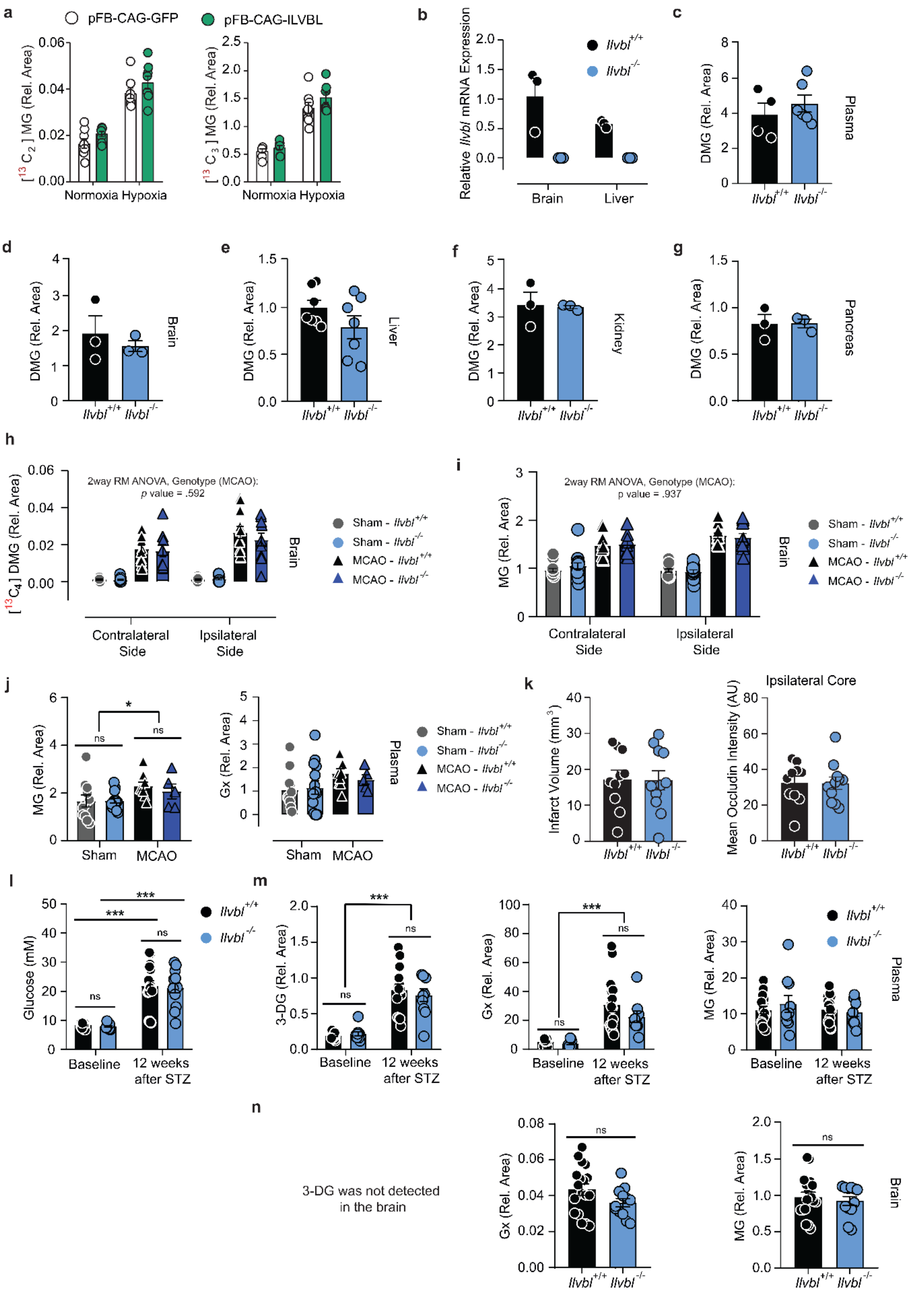

Supplementary Fig. 3 | ILVBL is not involved in the generation of 3-deoxyglucosone, glyoxal, or methylglyoxal. a [$^{13}C_2$]-methylglyoxal ([$^{13}C_2$]MG) and [$^{13}C_3$]MG in medium of control (pFB-CAG-GFP) or *IlvB* acetolactate synthase-like (ILVBL)-overexpressing cells, incubated for 6 h under normoxic or hypoxic conditions with [$^{13}C_6$]-glucose (*n*=8 per group). **b** Relative *Ilvbl* mRNA expression in brain and

liver of *Ilvbl*$^{+/+}$ and *Ilvbl*$^{-/-}$ mice (*n*=3 per group). **c-g** Dimethylglyoxal (DMG) in plasma (*n*=4 (*Ilvbl*$^{+/+}$), *n*=6 (*Ilvbl*$^{-/-}$)), brain (*n*=3 per group), liver (*n*=7 per group), kidney (*n*=3 per group), and pancreas (*n*=3 per group) of *Ilvbl*$^{+/+}$ and *Ilvbl*$^{-/-}$ mice under basal conditions. **h** [$^{13}C_4$]DMG in the ipsi- and contralateral hemispheres of *Ilvbl*$^{+/+}$ and *Ilvbl*$^{-/-}$ mice, 50 min after sham surgery (*n*=14 (*Ilvbl*$^{+/+}$), *n*=17 (*Ilvbl*$^{-/-}$)) or occlusion of the distal middle cerebral artery (MCAO, *n*=12 (*Ilvbl*$^{+/+}$), *n*=10 (*Ilvbl*$^{-/-}$)). **i** MG in the ipsi- and contralateral hemispheres of *Ilvbl*$^{+/+}$ and *Ilvbl*$^{-/-}$ mice, 50 min after sham surgery (*n*=14 (*Ilvbl*$^{+/+}$), *n*=17 (*Ilvbl*$^{-/-}$)) or MCAO (*n*=12 (*Ilvbl*$^{+/+}$), *n*=10 (*Ilvbl*$^{-/-}$)). **j** MG and glyoxal (Gx) in plasma of *Ilvbl*$^{+/+}$ and *Ilvbl*$^{-/-}$ mice, 50 min after sham surgery (*n*=11 (*Ilvbl*$^{+/+}$), *n*=14 (*Ilvbl*$^{-/-}$)) or MCAO (*n*=9 (*Ilvbl*$^{+/+}$), *n*=5 (*Ilvbl*$^{-/-}$)). **k** Quantification of infarct volumes and occludin intensity in ipsilateral hemisphere of *Ilvbl*$^{+/+}$ (*n*=10) and *Ilvbl*$^{-/-}$ (*n*=11) mice 48 hours after MCAO. AU, arbitrary units. **l** Blood glucose concentrations of *Ilvbl*$^{+/+}$ (*n*=18) and *Ilvbl*$^{-/-}$ (*n*=14) mice at baseline and 12 weeks after streptozotocin (STZ) treatment. **m** 3-Deoxyglucosone (3-DG), Gx, and MG in plasma of *Ilvbl*$^{+/+}$ (*n*=16) and *Ilvbl*$^{-/-}$ (*n*=9-10) mice at baseline and 12 weeks after STZ treatment. **n** Gx and MG in brain of *Ilvbl*$^{+/+}$ (*n*=18) and *Ilvbl*$^{-/-}$ (*n*=12) mice 12 weeks after STZ treatment. Values are means ± SEM. Statistical comparison by 2-way ANOVA (a ($^{13}C_2$ MG) and j (Gx)), 2-way RM ANOVA (h, i and m), 2-way ANOVA followed by Sidak´s post-hoc tests (l), Scheirer-Ray-Hare test (a ($^{13}C_3$ MG) and j (MG)) or two-tailed unpaired t test (c, d, e, f, g, k and n). $*p < .05$; $***p < .001$. Detailed information on the test statistics is provided in Supplementary Table 6.

Supplementary Fig. 4

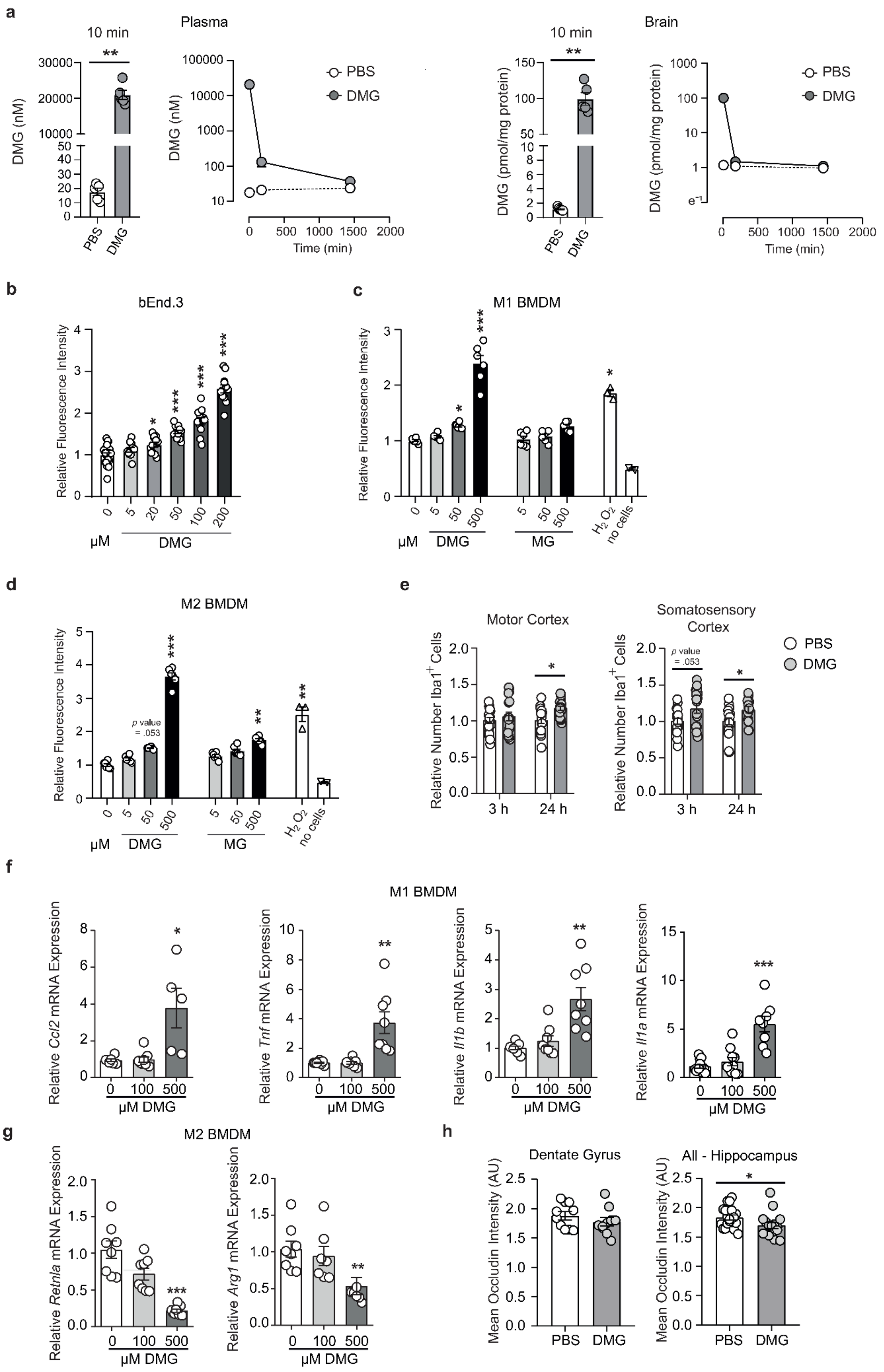

i

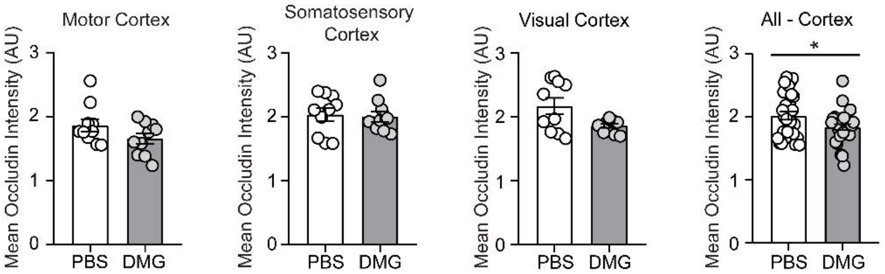

j

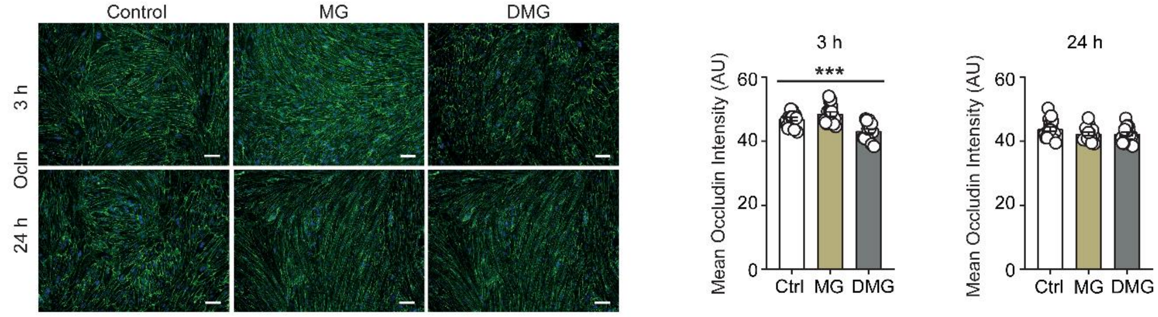

Supplementary Fig. 4 | Effects of dimethylglyoxal *in vivo* and *in vitro*. a Concentrations of dimethylglyoxal (DMG) in plasma and brain 10 min (*n*=5 per group), 3 h (Plasma – *n*=9 (PBS), *n*=11 (DMG); Brain – *n*=5 (PBS), *n*=4 (DMG)), and 24 h (Plasma – *n*=9 (PBS), *n*=10 (DMG); Brain – *n*=5 per group) after DMG injection. A base-10 log scale was used for the Y axes of right graphs. **b** Treatment of brain endothelial bEnd.3 cells with DMG for 10 min induced reactive oxygen species (ROS). ROS production was detected with the dye chloromethyl 2′,7′-dichlorodihydrofluorescein diacetate (CM-H_2DCFDA, *n*=24 for 0 µM, *n*=11-12 for ≥ 5 µM). **c, d** Induction of ROS in pro-inflammatory M1-polarized bone marrow-derived macrophages (M1 BMDM; c) and anti-inflammatory M2-polarized macrophages (M2 BMDM; d) by DMG, methylglyoxal (MG) or hydrogen peroxide (H_2O_2; 10 µM) for 10 min (*n*=2-6). **e** Quantification of Iba1$^+$ microglia and brain macrophages in motor cortex (*n*=17-18) and somatosensory cortex (*n*=17-18) 3 h and 24 h after single i.p. injection of 100 mg/kg DMG. **f, g** mRNA levels of pro- and anti-inflammatory genes in M1 BMDM (f, *n*=5-8 (*Ccl2)*, *n*=8 (*Tnf* and *Il1b)*, and *n*=8-12 (*Il1a)*) and M2 BMDM (g, *n*=8 (*Retnla)* and *n*=6-8 (*Arg1)*) after DMG treatment for 48 h. **h, i** Quantification of occludin (Ocln) intensity in brain vessels in the dentate gyrus, as well as motor, somatosensory and visual cortex 3 h after DMG injection (*n*=10 per group). AU, arbitrary units. **j** Representative immunocytochemical microscope images of Ocln in primary brain endothelial cells (PBEC) after 3 h or 24 h treatment with 500 µM methylglyoxal (MG) or 500 µM DMG and quantification of mean Ocln intensity (*n*= 16 per group). Values are means ± SEM. Statistical comparison by two-tailed Mann-Whitney U test (a (10 min), h (all hippocampus) and i (visual cortex)), one-way ANOVA followed by Dunnett´s post hoc tests (b and j), Kruskal-Wallis followed by Dunn´s post hoc tests (c, d, f and g), 2-way ANOVA followed by targeted Sidak´s post hoc tests (e (motor cortex)), Scheirer-Ray-Hare test followed by two-tailed Mann Whitney tests and Bonferroni-Holm correction (e (somatosensory cortex)) and two-tailed unpaired t test (h (dentate gyrus) and i (motor cortex, somatosensory cortex and all cortex)). **p* < .05; ***p* < .01; ****p* < .001. Scale bar 100 µm. Detailed information on the test statistics is provided in Supplementary Table 6.

Supplementary Fig. 5

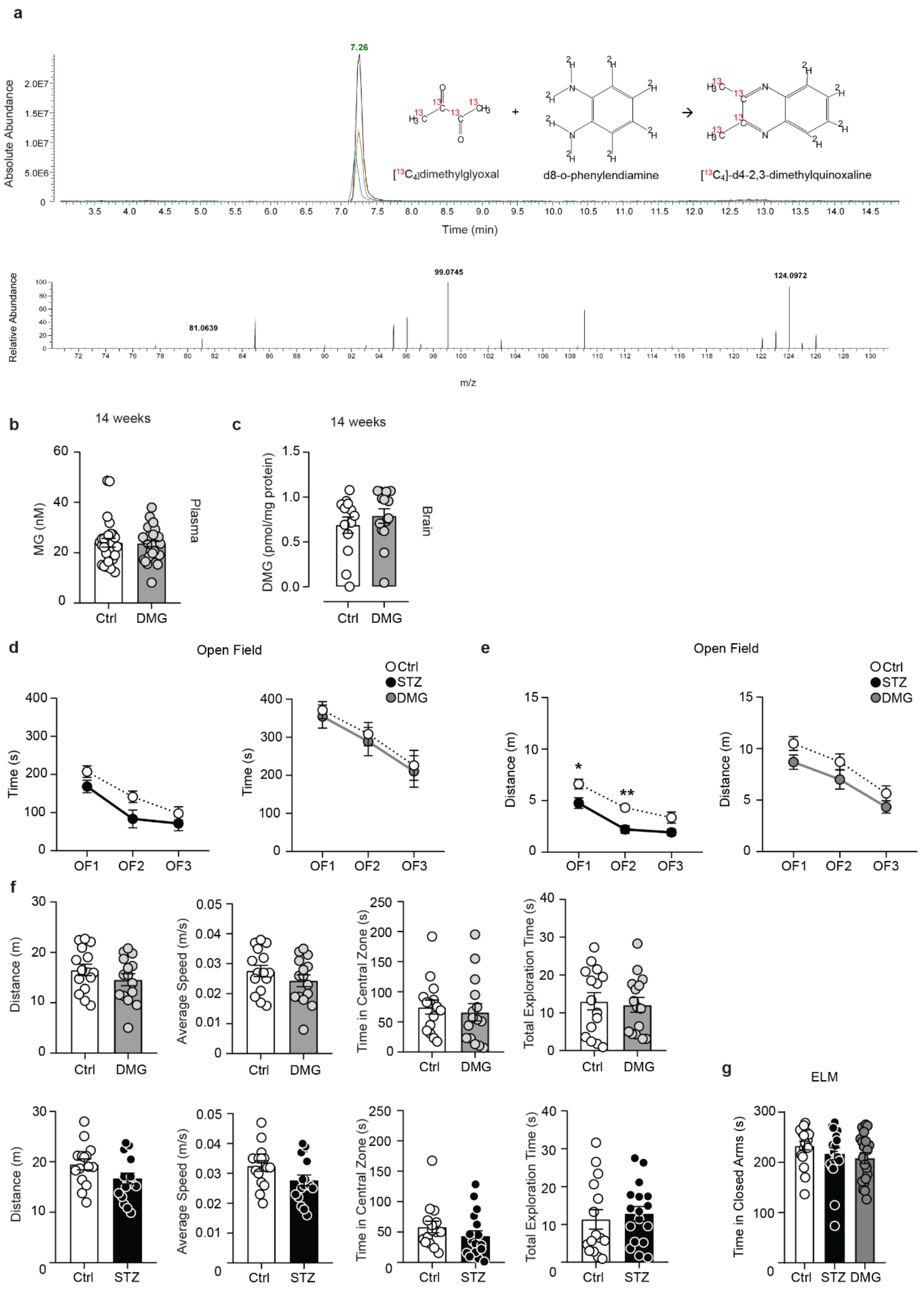

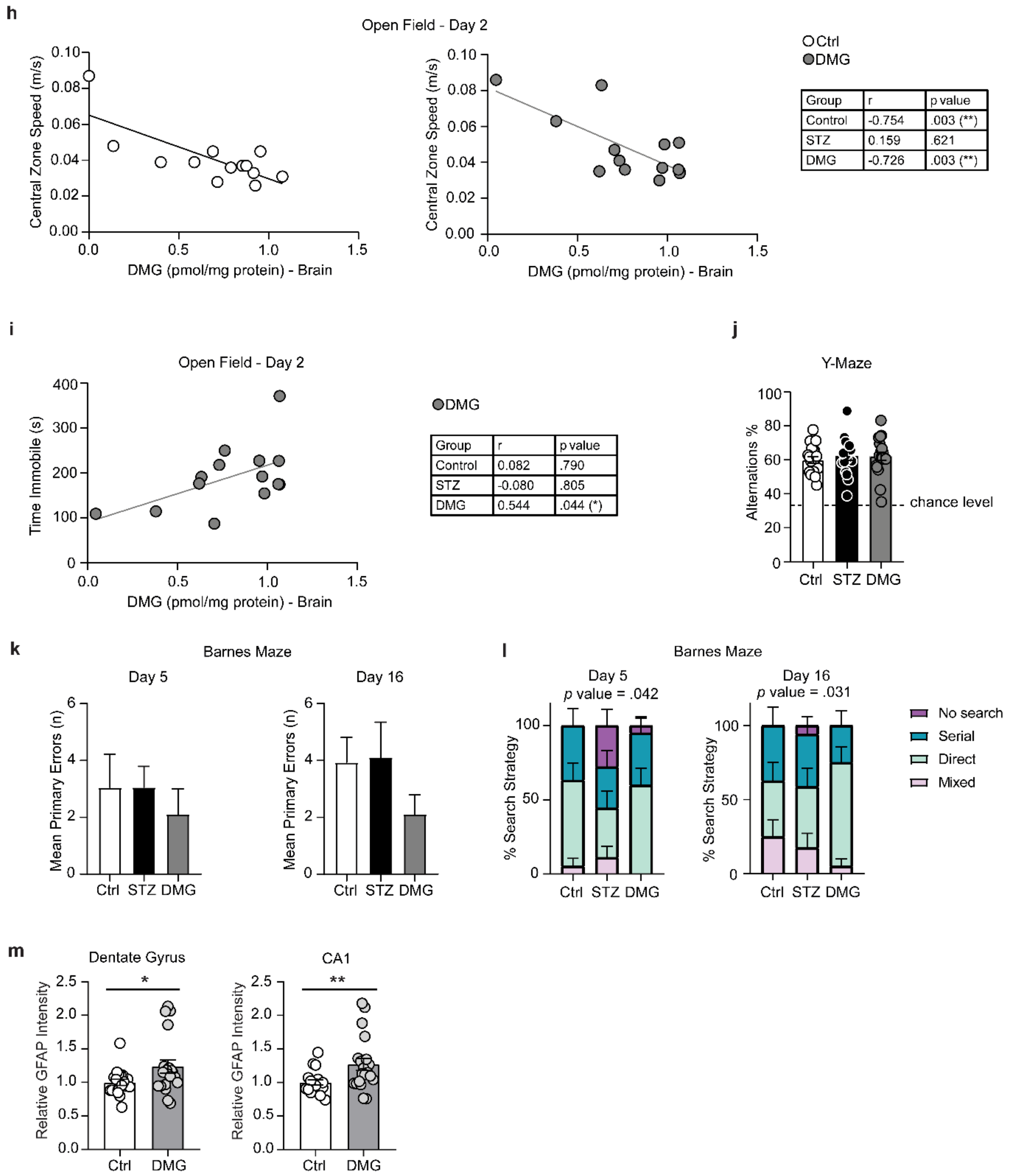

Supplementary Fig. 5 | Behavioral effects of dimethylglyoxal and STZ treatment. a Representative chromatogram and fragmentation mass spectrum of [$^{13}C_4$]-2,3-dimethylquinoxaline ([$^{13}C_4$]2,3-DMQ) (retention time = 7.26 min; *m/z* = 167.13020) in the plasma of a mouse treated with [$^{13}C_4$]-dimethylglyoxal ([$^{13}C_4$]DMG) (5 mg/kg) via oral gavage. **b** Plasma concentrations of methylglyoxal (MG) after treating mice for 14-15 weeks with dimethylglyoxal (DMG) in the drinking water (*n*=27-28). **c** Brain concentrations of DMG after treating mice for 14-15 weeks with DMG in the drinking water (*n*=13-14). **d, e** Mobility (time and distance) of mice treated with streptozotocin (STZ, *n*=15-17) or DMG (*n*=15 per group) in the drinking water for 14-15 weeks. Mice were tested in an open field. **f** Mobility (distance and average speed), anxiety-related behavior (time in central zone) and motivation (total exploration time) of mice after chronic DMG (*n*=15 per group) or STZ (*n*=15-17) treatment during the object place recognition test (OPRT). **g** Anxiety-related behavior of control (*n*=19), STZ-treated mice (*n*=19) or mice after chronic DMG treatment (*n*=20) in an elevated plus maze (ELM). **h, i** Correlation between central zone speed (*n*=13-14) or time immobile (*n*=14) and DMG concentrations in the brain in an open field (Day 2). **j** Alternation behavior and spatial short-term memory of control (*n*=20) STZ-treated mice (*n*=19)

or mice after chronic DMG treatment (*n*=20) in a Y-maze. **k, l** Spatial long-term memory of STZ-treated mice or mice after chronic DMG treatment in the Barnes maze at day 5 (*n*=18-20) and day 16 (*n*=16-20). Mean primary errors and the search strategy used are shown. **m** Quantification of mean glial fibrillary acidic protein (GFAP) immunohistological staining intensity in dentate gyrus and CA1 (*n*=19-20). Values are means ± SEM. Statistical comparison by two-tailed Mann-Whitney U test (b, c, f (time in central zone (DMG and STZ), and total exploration (STZ)) and m), mixed-effects analysis (REML, d) followed by Sidak´s post hoc tests (e), two-tailed unpaired t test (f (speed and distance (DMG and STZ)), total exploration (DMG)), Kruskal-Wallis test (g and k), Pearson correlation coefficient (h and i), one-way ANOVA (j) or Chi-squared test (l). *$p < .05$; **$p < .01$. Detailed information on the test statistics is provided in Supplementary Table 6.

Supplementary Table 1

Compound	Retention time (min)	Precursor ion (m/z)	Product ion (m/z)	Collision energy (V)
2,3-DMQ	7.20	159.10	77.23	32
			118.14	24
d4-MG	6.46	153.13	81.27	32
			125.10	24
Gx	5.50	135.09	81.20	30
			108.10	21
3-DG	3.05	239.13	81.20	18
			149.10	36
			203.10	26
MG	6.46	149.10	81.27	31
			122.16	23
DMG	7.14	163.12	81.27	36
			125.10	24
Pyruvate	6.58	165.09	96.07	35
			137.10	35
[$^{13}C_3$]-pyruvate	6.58	168.11	96.07	35
			139.10	35
d4-CEL	7.29	223.16	88.22	19
			134.10	10
HBL	7.58	233.15	84.10	25
			144.10	15

Masses of α-dicarbonyls refer to the respective d4-quinoxaline derivate.

Supplementary Table 2

Compound	^{12}C- or ^{13}C-labeled Ion (m/z)
Gx	135.08548
Gx M+1	136.08884
Gx M+2	137.09220
MG	149.10113
MG M+1	150.10449
MG M+2	151.10784
MG M+3	152.11120
DMG	163.11678
DMG M+1	164.12014
DMG M+2	165.12349
DMG M+3	166.12685
DMG M+4	167.13020
Pyruvate	165.09615
Pyruvate M+3	168.10616

Masses of α-dicarbonyls refer to the respective d4-quinoxaline derivate.

Supplementary Table 3

Gene	Direction	Sequence
Ppia	forward	5’-AGGTCCTGGCATCTTGTCCAT- 3’
	reverse	5’-GAACCGTTTGTGTTTGGTCCA- 3’
Ccl2	forward	5’-GCTCAGCCAGATGCAGTTAACGC- 3’
	reverse	5’-GCTTCTTTGGGACACCTGCTGCT- 3’
Tgfb2	forward	5’-CTTCGACGTGACAGACGCT- 3’
	reverse	5’-GCAGGGGCAGTGTAAACTTATT- 3’
Retnla	forward	5’-TCCTGCCCTGCTGGGATGACTGCTA- 3’
	reverse	5’-CAGCGGGCAGTGGTCCAG TCAA- 3’
Arg1	forward	5’-TGGTGTGGTGGCAGAGGTCCA- 3’
	reverse	5’-ACTGCCAGACTGTGGTCTCCACC- 3’
Tnf	forward	5’-TGTAGCCCACGTCGTAGCAAA- 3’
	reverse	5’-GCTGGCACCACTAGTTGGTTGT- 3’
Il1b	forward	5’-CTGTGACTCATGGGATGATGATG-3’
	reverse	5’-GGAGCCTGTAGTGCAGTTG-3’
Il1a	forward	5’-GATCTGGAAGAGACCAT-3’
	reverse	5’-CTGGCAACTCCTTCAG-3’
Ilvbl	forward	5‘-CCCAGGTCCAGTGTTTGTGG-3‘
	reverse	5‘-ACGGCAGCTCTAAGCTTGTT-3‘
Pla2g4a	forward	5‘-GAAGGCACAGAGAAGCCTGAG-3‘
	reverse	5‘-ATGCTGAACCGTAGGTCTGG-3‘
Ptges	forward	5‘-CTTGCGGACTTGGTGTCTCT-3‘
	reverse	5‘-GCACTCAAAGGACGGTGGTA-3‘
Ptgs2	forward	5‘-CATCCCCTTCCTGCGAAGTT-3‘
	reverse	5‘-CATGGGAGTTGGGCAGTCAT-3‘
Bim	forward	5‘-ACAGAACCGCAAGCTTCCAT-3‘
	reverse	5‘-CAGATCTTCAGGTTCCTCCTGAGA-3‘

Supplementary Table 4

Baseline Characteristic	Patients with diabetes (n=100)	Patients without diabetes (n=100)
Diabetes type		
1	13/100 (13)	-
2	77/100 (77)	-
NC	10/100 (10)	-
Age (y), (year, 95% CI)	59.0 (56.27-62.48)	59.5 (56.34-62.46)
Women (%)	36/100 (36)	37/100 (37)
Glucose (mmol/L, 95% CI)	10.57 (10.5-13.45)	4.59 (4.72-5.69)
Body weight (kg, 95% CI)	86.5 (79.2-110.4)	84.0 (80.8-88.6)

Values are median (95 % CI) or n/total (%) as appropriate.

Supplementary Table 5

Figure	Sample size (n)	Statistical test	Values	Comments
1b	**Control:** 15 mice<br>**STZ:** 17 mice	Two-tailed Mann-Whitney U test	$p = 4 \times 10^{-9}$	
1c	8 mice/group	Two-tailed Mann-Whitney U test	$p = 2 \times 10^{-4}$	
1e	**3-DG:** 15 mice (Control) and 16 mice (STZ)<br>**Gx:** 15 mice (Control) and 16 mice (STZ)<br>**MG:** 15 mice (Control) and 17 mice (STZ)<br>**DMG:** 15 mice/group	**3-DG**, **Gx** and **MG**: Two-tailed unpaired t test<br>**DMG:** Two-tailed Mann-Whitney U test	**3-DG:** T (29) = 5.1, $p = 2 \times 10^{-5}$<br>**Gx:** T (29) = 1.4, p = .170<br>**MG:** T (30) = 1.1, p = .260<br>**DMG:** p = .004	Values excluded:<br>**3-DG:** 1 outlier (STZ)<br>**Gx:** 1 outlier (STZ)<br>**DMG:** 2 outliers (STZ)
1f	**3-DG:** 6 mice (Control) and 11 mice (STZ)<br>**Gx:** 14 mice (Control) and 15 mice (STZ)<br>**MG:** 14 mice (Control) and 16 mice (STZ)<br>**DMG:** 14 mice (Control) and 17 mice (STZ)	Two-tailed unpaired t test	**3-DG:** T (15) = 0.9, p = .376<br>**Gx:** T (27) = 1.9, p = .070<br>**MG:** T (28) = 2.3, p = .030<br>**DMG:** T (29) = 2.6, p = .014	Values excluded:<br>**3-DG:** 9 values in Control group and 5 values in STZ group were excluded because the relative area was lower than LOD; 1 outlier (STZ)<br>**Gx:** 1 outlier (Control) and 1 outlier (STZ)
1g	**3-DG:** 11 mice (db/+) and 9 mice (db/db)<br>**Gx, MG and DMG**: 11 mice (db/+) and 10 mice (db/db)	Two-tailed Mann-Whitney U test	**3-DG:** p = .006<br>**Gx:** p > .999<br>**MG:** $p = 4 \times 10^{-4}$<br>**DMG:** p = .004	Value excluded: 1 db/db mouse because of lack of hyperglycemia
2a	**Glucose:** 100 patients/group<br>**3-DG:** 100 patients (Control) and 99 patients (Diabetes)<br>**DMG:** 100 patients (Control) and 99 patients (Diabetes)<br>**MG:** 100 patients (Control) and 98 patients (Diabetes)<br>**Gx:** 100 patients (Control) and 99 patients (Diabetes)	Two-tailed Mann-Whitney U test	**Glucose:** $p < 1 \times 10^{-15}$<br><br>**3-DG:** $p = 4 \times 10^{-13}$<br>**Gx:** p = .001<br>**MG:** $p = 2 \times 10^{-4}$<br>**DMG:** $p = 2 \times 10^{-7}$	Values excluded:<br>**3-DG**: 1 outlier (Diabetes)<br>**DMG**: 1 outlier (Diabetes)<br>**MG**: 2 outliers (Diabetes)<br>**Gx**: 1 outlier (Diabetes)
2b	**Glucose:**<br>Control: 14 patients (Type 1), 77 patients (Type 2) and 8 patients (Non classified)<br>Diabetes: 13 patients (Type 1), 77 patients (Type 2) and 7	Scheirer-Ray-Hare test followed by two-tailed Mann-Whitney U post hoc tests, Bonferroni-Holm correction	**Glucose:**<br>Diabetes: χ^2 (1) = 38.9, $p = 4 \times 10^{-10}$<br>Type: χ^2 (2) = 1.0, p = .622<br>Interaction: χ^2 (2) = 1.0, p = .610	

	patients (Non classified)<br><br>**3-DG:**<br>Control: 14 patients (Type 1), 77 patients (Type 2) and 8 patients (Non classified)<br>Diabetes: 13 patients (Type 1), 75 patients (Type 2) and 7 patients (Non classified)<br><br>**Gx:**<br>Control: 14 patients (Type 1), 77 patients (Type 2) and 8 patients (Non classified)<br>Diabetes: 13 patients (Type 1), 76 patients (Type 2) and 7 patients (Non classified)<br><br>**MG:**<br>Control: 14 patients (Type 1), 77 patients (Type 2) and 8 patients (Non classified)<br>Diabetes: 13 patients (Type 1), 75 patients (Type 2) and 7 patients (Non classified)<br><br>**DMG:**<br>Control: 14 patients (Type 1), 77 patients (Type 2) and 8 patients (Non classified)<br>Diabetes: 13 patients (Type 1), 76 patients (Type 2) and 7 patients (Non classified)		Type 1 (Control vs Diabetes): $p = 2 \times 10^{-4}$<br>Type 2 (Control vs Diabetes): $p = .002$<br>Non classified (Control vs Diabetes): $p = .006$<br><br>**3-DG:**<br>Diabetes: χ^2 (1) = 27.7, $p = 1.4 \times 10^{-7}$<br>Type: χ^2 (2) = 3.6, $p = .162$<br>Interaction: χ^2 (2) = 2.1, $p = .354$<br><br>Type 1 (Control vs Diabetes): $p = 6 \times 10^{-4}$<br>Type 2 (Control vs Diabetes): $p = 3 \times 10^{-4}$<br>Non classified (Control vs Diabetes): $p = .152$<br><br>**Gx:**<br>Diabetes: χ^2 (1) = 3.9, $p = .048$<br>Type: χ^2 (2) = 13.1, $p = .001$<br>Interaction: χ^2 (2) = 1.4, $p = .499$<br><br>Type 1 (Control vs Diabetes): $p = .651$<br>Type 2 (Control vs Diabetes): $p = 3 \times 10^{-4}$<br>Non classified (Control vs Diabetes): $p = .867$<br><br>**MG:**<br>Diabetes: χ^2 (1) = 4.2, $p = .039$<br>Type: χ^2 (2) = 2.9, $p = .234$<br>Interaction: χ^2 (2) = 0.7, $p = .714$<br><br>Type 1 (Control vs Diabetes): $p = .917$<br>Type 2 (Control vs Diabetes): $p = 3 \times 10^{-4}$<br>Non classified (Control vs Diabetes): $p = .612$	

			**DMG:**<br>Diabetes: χ^2 (1) = 13.4, p = 3 × 10^{-4}<br>Type: χ^2 (2) = 1.9, p = .395<br>Interaction: χ^2 (2) = 0.4, p = .811<br><br>Type 1 (Control vs Diabetes): p = .034<br>Type 2 (Control vs Diabetes): p = .003<br>Non classified (Control vs Diabetes): p = .189	
2c	**Glucose, 3-DG and DMG:** 33 patients (Insulin), 19 patients (Metformin) and 8 patients (Insulin + Metformin)<br><br>**Gx and MG:** 32 patients (Insulin), 19 patients (Metformin) and 8 patients (Insulin + Metformin)	Kruskal-Wallis followed by Dunn´s post hoc tests (*p*-values adjusted for multiple comparisons)	**Glucose:** χ^2 (2) = 1.1, p = .587<br><br>**3-DG:** χ^2 (2) = 8.7, p = .013<br><br>Insulin vs Metformin: p > .999<br>Insulin vs Insulin + Metformin: p = .029<br>Metformin vs Insulin + Metformin: p = .012<br><br>**Gx:** χ^2 (2) = 6.3, p = .044<br><br>Insulin vs Metformin: p > .999<br>Insulin vs Insulin + Metformin: p = .046<br>Metformin vs Insulin + Metformin: p = .078<br><br>**MG:** χ^2 (2) = 2.2, p = .336<br><br>**DMG:** χ^2 (2) = 15.5, p = 4 × 10^{-4}<br><br>Insulin vs Metformin: p = .119<br>Insulin vs Insulin + Metformin: p = 5 × 10^{-4}<br>Metformin vs Insulin + Metformin: p = .101	
3b	10 mice/group	Scheirer-Ray-Hare test followed by two-tailed Mann-Whitney U post hoc tests, Bonferroni-Holm correction	**Day 10:**<br>Diet: χ^2 (1) = 0.15, p = .700<br>Treatment: χ^2 (1) = 22.7, p = 2 × 10^{-6}<br>Interaction: χ^2 (1) = 0.7, p = .410	

			Vehicle-chow vs. STZ-chow: *p* = .001<br>Vehicle-KD vs. STZ-KD: *p* = 2×10^{-4}<br><br>**Day 56:**<br>Diet: χ^2 (1) = 4.9, *p* = .028<br>Treatment: χ^2 (1) = 13.3, *p* = 3×10^{-4}<br>Interaction: χ^2 (1) = 0.3, *p* = .600<br><br>Vehicle-chow vs. STZ-chow: *p* = .002<br>Vehicle-KD vs. STZ-KD: *p* = .006<br>Vehicle-chow vs. Vehicle-KD: *p* = .171<br>STZ-chow vs. STZ-KD: *p* = .014	
3c	**Vehicle:** 10 mice/group<br>**STZ:** 9 mice (Chow) and 10 mice (KD)	2-way ANOVA followed by Sidak´s post hoc tests (*p*-values adjusted for multiple comparisons)	Diet**:** *F* (1,35) = 0.7, *p* = .412 Treatment**:** *F* (1,35) = 15.6, *p* <4×10^{-4}<br>Interaction**:** *F* (1,35) = 0.4, *p* = .513<br><br>Vehicle-chow vs STZ-chow: *p* = .006<br>Vehicle-KD vs STZ-KD: *p* = .048	
3d	**Vehicle**: 7 mice (Chow) and 7 mice (KD, 6 for 3-DG and DMG)<br>**STZ:** 8 mice (Chow) and 7 mice (KD, 6 for MG and DMG)	**3-DG and DMG:** Scheirer-Ray-Hare test followed by two-tailed Mann-Whitney U post hoc tests, Bonferroni-Holm correction<br><br>**Gx** and **MG**: 2-way ANOVA	**3-DG:**<br>Diet: χ^2 (1) = 7.0, *p* = .008<br>Treatment: χ^2 (1) = 1.1, *p* = .295<br>Interaction: χ^2 (1) = 5.3, *p* = .021<br><br>Vehicle-chow vs. STZ-chow: *p* = .018<br>Vehicle-KD vs. STZ-KD: *p* = .890<br>Vehicle-chow vs. Vehicle-KD: *p* = .890<br>STZ-chow vs. STZ-KD: *p* = .001<br><br>**Gx:**<br>Diet: *F* (1,25) < 0.01, *p* = .948<br>Treatment: *F* (1,25) < 0.01, *p* = .947<br>Interaction: *F* (1,25) = 0.5, *p* = .471<br><br>**MG:**	Value excluded: 1 outlier for MG (STZ, KD)

			Diet: F (1,24) = 3.7, p = .065<br>Treatment: F (1,24) = 0.04, p = .842<br>Interaction: F (1,24) = 0.08, p = .774<br><br>**DMG:**<br>Diet: χ^2 (1) = 15.4, p = 9 × 10^{-5}<br>Treatment: χ^2 (1) = 3.1, p = .076<br>Interaction: χ^2 (1) = 0.06, p = .807<br><br>Vehicle-chow vs. STZ-chow: p = .058<br>Vehicle-KD vs. STZ-KD: p = .132<br>Vehicle-chow vs. Vehicle-KD: p = .004<br>STZ-chow vs. STZ-KD: p = .003	
4a	3 wells/group	**3-DG:** Descriptive only<br>**Gx**: Two-tailed unpaired t test<br>**MG:** Descriptive only/two-tailed unpaired t test<br>**DMG:** Descriptive only/two-tailed unpaired t test	**Gx:**<br>$^{13}C_1$: T (4) = 0.7, p = .535<br>$^{13}C_2$: T (4) = 29.9, p = 8 × 10^{-6}<br><br>**MG:**<br>$^{13}C_1$: T (4) = 0.5, p = .662<br>$^{13}C_2$: T (4) = 11.6, p = 3 × 10^{-4}<br><br>**DMG:**<br>$^{13}C_1$: T (4) = 0.7, p = .547<br>$^{13}C_2$: T (4) = 7.9, p = .001	
4b	8 wells/group (360 min)	Descriptive only/Two-tailed Mann-Whitney U test	p = 2 × 10^{-4}	
4c	8 wells/group (360 min)	Descriptive only/Two-tailed unpaired t test	T (14) = 4.1, p = .001	
4e	**Vehicle:** 7 mice/group<br>**Glucose:** 8 mice/group	2-way RM ANOVA followed by Sidak´s post hoc tests (p-values adjusted for multiple comparisons)	**30 min:** Time: F (1,13) = 9.8, p = .008 Treatment: F (1,13) = 0.9, p = .355<br>Interaction: F (1,13) = 2.6, p = .128<br>Baseline vs Vehicle-30 min: p = .539<br>Baseline vs Glucose-30 min: p = .008<br><br><br>**50 min:**	Values excluded:<br>**50 min:** 1 outlier (Vehicle, value was also excluded at 0 min)

			Time: *F* (1,13) = 104.8, $p = 1 \times 10^{-7}$<br>Treatment: *F* (1,13) = 9.1, *p* = .010<br>Interaction: *F* (1,13) = 4.4, *p* = .056<br>Vehicle-50 min vs Glucose-50 min: *p* = .009<br>Baseline vs Vehicle-50 min: *p* = .0002<br>Baseline vs Glucose-50 min: $p = 1 \times 10^{-6}$	
4f	**Vehicle:** 7 mice (30 min, both hemispheres) and 5 ipsi- and 6 contralateral hemisphere (50 min)<br>**Glucose:** 8 mice (30 min, both hemispheres) and 8 ipsi- and 7 contralateral hemisphere (50 min)	Mixed-effects analysis (REML) followed by Sidak´s post hoc tests (*p*-values adjusted for multiple comparisons)	**30 minutes:**<br>Hemisphere: *F* (1,26) = 0.6, *p* = .433<br>Treatment: *F* (1,26) = 0.02, *p* = .876<br>Interaction: *F* (1,26) = 0.1, *p* = .714<br><br>**50 minutes:**<br>Hemisphere: *F* (1,24) = 7.1, *p* = .013<br>Treatment: *F* (1,24) = 0.1, *p* = .783<br>Interaction: *F* (1,24) = 2.3, *p* = .144<br><br>Ipsilateral hemisphere-Vehicle vs Contralateral hemisphere-Vehicle: *p* = .682<br><br>Ipsilateral hemisphere-Glucose vs Contralateral hemisphere-Glucose: *p* = .011	Values excluded:<br>**50 min - Vehicle:** 2 values (hemispheres from same mouse) were excluded because this mouse was also excluded in Fig.4e
5a	12 wells/group	Kruskal-Wallis followed by Dunn´s post hoc tests (*p*-values adjusted for multiple comparisons)/Descriptive only	**$^{13}C_2$:** χ^2 (4) = 20.0, $p = 5 \times 10^{-4}$<br><br>^{12}C vs 1-$^{13}C_1$: *p* > .999<br>^{12}C vs 2-$^{13}C_1$: *p* = .001<br>^{12}C vs 3-$^{13}C_1$: *p* = .007<br>^{12}C vs 1,2,3-$^{13}C_3$: *p* = .028	
5c	**Cell-free:** 4 wells/group<br>**bEnd.3 cells:** 10 wells/group	Scheirer-Ray-Hare test followed by two-tailed Mann-Whitney U post hoc tests, Bonferroni-Holm correction	Group: χ^2 (1) = 16.6, $p = 5 \times 10^{-5}$<br>Oxygen: χ^2 (1) = 3.3, *p* = .069	

			Interaction: χ^2 (1) = 1.2, *p* = .275<br><br>Cell-free-Normoxia vs. Cell-free-Hypoxia: *p* = .200<br>bEnd.3 cells-Normoxia vs. bEnd.3 cells-Hypoxia: *p* = 4 × 10^{-5}<br>Cell-free-Normoxia vs. bEnd.3 cells-Normoxia: *p* = .006<br>Cell-free-Hypoxia vs. bEnd.3 cells-Hypoxia: *p* = .004	
5e	6 wells/group	Scheirer-Ray-Hare test followed by two-tailed Mann-Whitney U post hoc tests, Bonferroni-Holm correction	Transfection: χ^2 (1) = 17.3, *p* = 3 × 10^{-5}<br>Oxygen: χ^2 (1) = 1.9, *p* = .166<br>Interaction: χ^2 (1) = 0.5, *p* = .488<br><br>Control-GFP vs Control- ILVBL: *p* = .004<br>Hypoxia-GFP vs Hypoxia-ILVBL: *p* = .004	
5f	**Control (GFP):** 8 wells<br>**ILVBL overexpressed cells:** 7 wells	Two-tailed Mann-Whitney U test	*p* = 3 × 10^{-4}	
5g	**DMG (M+2):** 8 wells/group<br>**DMG (M+4):** 7 wells (GFP-Hypoxia) and 8 wells (other groups)	2-way ANOVA followed by Tukey´s post-hoc tests (*p*-values adjusted for multiple comparisons)	**DMG (M+2):**<br>Transfection: *F* (1,28) < .001, *p* = .992<br>Oxygen: *F* (1,28) = 0.7, *p* = .425<br>Interaction: *F* (1,28) = 0.02, *p* = .881<br><br>**DMG (M+4):**<br>Transfection: *F* (1,27) = 7.4, *p* = .011<br>Oxygen: *F* (1,27) = 46.8, *p* = 2 × 10^{-7}<br>Interaction: *F* (1,27) = 3.9, *p* = .058<br><br>Control-GFP vs Control- ILVBL: *p* = .839<br>Hypoxia-GFP vs Hypoxia-ILVBL: *p* = .006	
6b	**Sham – *Ilvbl*$^{+/+}$:** 14 mice	2-way RM ANOVA followed by Sidak´s post	**MCAO:**<br>**DMG (M):**	

	**Sham – *Ilvbl*$^{-/-}$:** 17 mice<br>**MCAO – *Ilvbl*$^{+/+}$:** 12 mice<br>**MCAO – *Ilvbl*$^{-/-}$:** 10 mice	hoc tests (*p*-values adjusted for multiple comparisons)	Side: F (1,20) = 27.65, p = 4 × 10^{-5}<br>Genotype: F (1,20) = 7.4, p = .013<br>Interaction: F (1,20) = 2.3, p = .145<br><br>Contralateral-*Ilvbl*$^{+/+}$ vs Contralateral-*Ilvbl*$^{-/-}$: p = .075<br>Ipsilateral-*Ilvbl*$^{+/+}$ vs Ipsilateral- *Ilvbl*$^{-/-}$: p = .008<br><br>**DMG (M+2):**<br>Side: F (1,20) = 21.70, p = 2 × 10^{-4}<br>Genotype: F (1,20) = 3.9, p = .063<br>Interaction: F (1,20) = 2.7, p = .117<br><br>Contralateral-*Ilvbl*$^{+/+}$ vs Contralateral-*Ilvbl*$^{-/-}$: p = .239<br>Ipsilateral-*Ilvbl*$^{+/+}$ vs Ipsilateral- *Ilvbl*$^{-/-}$: p = .056<br><br>**Sham:**<br>**DMG (M):**<br>Side: F (1,29) = 7.0, p = .013<br>Genotype: F (1,29) = 0.2, p = .628<br>Interaction: F (1,29) = 0.2, p = .672<br><br>**DMG (M+2):**<br>Side: F (1,29) = 1.5, p = .229<br>Genotype: F (1,29) = 1.8, p = .190<br>Interaction: F (1,29) = 0.05, p = .835	
6c	**Sham – *Ilvbl*$^{+/+}$:** 11 mice<br>**Sham – *Ilvbl*$^{-/-}$:** 14 mice<br>**MCAO – *Ilvbl*$^{+/+}$:** 9 mice<br>**MCAO – *Ilvbl*$^{-/-}$:** 5 mice	Scheirer-Ray-Hare test followed by two-tailed Mann-Whitney U post hoc tests, Bonferroni-Holm correction	**DMG (M):**<br>Treatment: χ^2 (1) = 2.7, p = .100<br>Genotype: χ^2 (1) = 1.0, p = .310<br>Interaction: χ^2 (1) = 0.3, p = .580<br><br>**DMG (M+2):**<br>Treatment: χ^2 (1) = 14.8, p = 1 × 10^{-4}<br>Genotype: χ^2 (1) = 1.6, p = .201<br>Interaction: χ^2 (1) = 0.5, p = .480	

			Sham-*Ilvbl*$^{+/+}$ vs MCAO-*Ilvbl*$^{+/+}$: p = .002<br>Sham- *Ilvbl*$^{-/-}$ vs MCAO-*Ilvbl*$^{-/-}$: p = .014<br><br>**DMG (M+4):**<br>Treatment: χ^2 (1) = 11.7, $p = 6 \times 10^{-4}$<br>Genotype: χ^2 (1) = 2.3 p = .131<br>Interaction: χ^2 (1) = 0.6, p = .445<br><br>Sham-*Ilvbl*$^{+/+}$ vs MCAO-*Ilvbl*$^{+/+}$: p = .004<br>Sham- *Ilvbl*$^{-/-}$ vs MCAO-*Ilvbl*$^{-/-}$: p = .070	
6e	***Ilvbl*$^{+/+}$:** 16 mice<br>***Ilvbl*$^{-/-}$:** 10 mice	2-way RM ANOVA	Time: F (1,24) = 9.4, p = .005<br>Genotype: F (1,24) = 0.5, p = .494<br>Interaction: F (1,24) = 0.7, p = .406	
6f	***Ilvbl*$^{+/+}$:** 18 mice<br>***Ilvbl*$^{-/-}$:** 12 mice	Two-tailed Mann-Whitney U test	p = .003	
7b	**Plasma:**<br>3 hours: 10 mice/group<br>24 hours: 9 mice (PBS) and 10 (DMG)<br><br>**Brain:**<br>3 hours: 5 mice (PBS) and 4 mice (DMG)<br>24 hours: 5 mice/group	**Plasma:**<br>Scheirer-Ray-Hare test followed by two-tailed Mann-Whitney U post hoc tests, Bonferroni-Holm correction<br><br>**Brain:**<br>2-way ANOVA followed by Tukey´s post-hoc tests (p-values adjusted for multiple comparisons)	**Plasma:**<br>Time: χ^2 (1) = 0.5, p = .472<br>Treatment: χ^2 (1) = 14.1 $p = 2 \times 10^{-4}$<br>Interaction: χ^2 (1) = 0.1, p = .701<br><br>Vehicle-3h vs DMG-3h: p = .011<br>Vehicle-24h vs DMG-24h: p = .011<br><br>**Brain:**<br>Time: F (1,15) = 17.0, $p = 9 \times 10^{-4}$<br>Treatment: F (1,15) = 17.9, $p = 7 \times 10^{-4}$<br>Interaction: F (1,15) = 4.0, p = .065<br><br>Vehicle-3 h vs DMG-3 h: p = .004<br>Vehicle-24 h vs DMG-24 h: p = .549<br>Vehicle-3 h vs Vehicle-24 h: p = .599<br>DMG-3 h vs DMG-24 h: p = .005	
7d	***Ilvbl*$^{+/+}$:** 14 mice<br>***Ilvbl*$^{-/-}$:** 17 mice	Two-tailed unpaired t test	T (29) = 2.4, p = .025	

8a	24 wells (0 µM DMG, 5 µM DMG, 50 µM DMG, 5 µM MG, 50 µM MG and 500 µM MG), 22 wells (500 µM DMG), 16 wells (10 µM H_2O_2) and 10 wells (no cells)	Kruskal-Wallis followed by Dunn´s post hoc tests (*p*-values adjusted for multiple comparisons)	χ^2 (8) = 108.7, $p = 1 \times 10^{-15}$<br><br>0 µM vs 5 µM DMG, $p > .999$<br>0 µM vs 50 µM DMG, $p = 6 \times 10^{-5}$<br>0 µM vs 500 µM DMG, $p = 1 \times 10^{-13}$<br><br>0 µM vs 5 µM MG, $p > .999$<br>0 µM vs 50 µM MG, $p = .117$<br>0 µM vs 500 µM MG, $p = .009$<br>0 µM vs 10 µM H_2O_2, $p = .003$<br>0 µM vs no cells, $p = .154$	
8b	3 wells (0 µM DMG, 5 µM DMG, 50 µM DMG, 500 µM DMG, 5 µM MG, 50 µM MG and 500 µM MG) and 2 wells (10 µM H_2O_2 and no cells)	Kruskal-Wallis followed by Dunn´s post hoc tests (*p*-values adjusted for multiple comparisons)	χ^2 (8) = 21.6, $p = .006$<br><br>0 µM vs 5 µM DMG, $p > .999$<br>0 µM vs 50 µM DMG, $p = .137$<br>0 µM vs 500 µM DMG, $p = .018$<br>0 µM vs 5 µM MG, $p > .999$<br>0 µM vs 50 µM MG, $p = .963$<br>0 µM vs 500 µM MG, $p > .999$<br>0 µM vs 10 µM H_2O_2, $p = .051$<br>0 µM vs no cells, $p > .999$	
8c	16 wells (0 µM DMG), 8 wells (5 µM DMG, 50 µM DMG and 500 µM DMG) and 12 wells (10 µM H_2O_2 and no cells))	Kruskal-Wallis followed by Dunn´s post hoc tests (*p*-values adjusted for multiple comparisons)	χ^2 (6) = 53.1, $p = 3 \times 10^{-10}$<br>0 µM vs 5 µM DMG, $p > .999$<br>0 µM vs 50 µM DMG, $p > .999$<br>0 µM vs 500 µM DMG, $p = 1 \times 10^{-4}$<br>0 µM vs 10 µM H_2O_2, $p = .003$<br>0 µM vs no cells, $p = .043$	
8e	**PBS-3 h:** 18 sections<br>**PBS-24 h:** 16 sections<br>**DMG-3 h:** 17 sections<br>**DMG-24 h:** 18 sections<br><br>3 sections per mouse (6 mice)	Scheirer-Ray-Hare test followed by two-tailed Mann-Whitney U post hoc tests, Bonferroni-Holm correction	Time: χ^2 (1) = 0.42, $p = .517$<br>Treatment: χ^2 (1) = 14.7 $p = 1 \times 10^{-4}$<br>Interaction: χ^2 (1) = 0.01, $p = .908$<br><br>Vehicle-3 h vs DMG-3 h: $p = .010$	

			Vehicle-24 h vs DMG-24 h: p = .002	
8f	***Ccl2*:**<br>10 wells (0 µM DM), 9 wells (100 µM DMG) and 8 wells (500 µM)<br>***Tgfb2*:**<br>12 wells (0 µM DMG) and 10 wells (100 µM and 500 µM)	***Ccl2*:**<br>Kruskal-Wallis followed by Dunn´s post hoc tests (p-values adjusted for multiple comparisons)<br><br>***Tgfb2*:**<br>One-way ANOVA followed by Dunnett´s post-hoc tests (p-values adjusted for multiple comparisons)	***Ccl2*:**<br>χ^2 (2) = 14.5, $p = 7 \times 10^{-4}$<br><br>0 µM vs 100 µM DMG: p = .060<br>0 µM vs 500 µM DMG: $p = 3 \times 10^{-4}$<br><br>***Tgfb2*:**<br>F (2,29) = 25.24, $p = 4 \times 10^{-7}$<br>0 µM vs 100 µM DMG: $p = 1 \times 10^{-4}$<br>0 µM vs 500 µM DMG: $p < 1 \times 10^{-4}$	Values excluded:<br>***Ccl2*:**<br>1 outlier in 0 µM DMG and 2 outliers in 100 µM DMG<br><br>***Tgfb2*:**<br>1 outlier in 100 µM DMG and 1 outlier in 500 µM DMG
8g	5 wells/group	***Pla2g4a*** and ***Ptges***:<br>One-way ANOVA followed by Dunnett´s post-hoc tests (p-values adjusted for multiple comparisons)<br><br>***Ptgs2*** and ***Bim***: Kruskal-Wallis followed by Dunn´s post hoc tests (p-values adjusted for multiple comparisons)	***Pla2g4a:***<br>F (2,12) = 7.15, p = .009<br><br>0 µM vs 100 µM DMG: p = .429<br>0 µM vs 500 µM DMG: p = .006<br><br>***Ptges:***<br>F (2,12) = 87.44, $p = 7 \times 10^{-8}$<br><br>0 µM vs 100 µM DMG: p = .001<br>0 µM vs 500 µM DMG: $p < 1 \times 10^{-4}$<br><br>***Ptgs2*:**<br>χ^2 (2) = 12.0, $p = 2 \times 10^{-5}$<br><br>0 µM vs 100 µM DMG: p = .207<br>0 µM vs 500 µM DMG: p = .001<br><br>***Bim*:**<br>χ^2 (2) = 8.4, p = .007<br><br>0 µM vs 100 µM DMG: p > .999<br>0 µM vs 500 µM DMG: p = .017	
8i	**PBS:** 9 mice/group<br>**DMG:** 6 mice (3 h) and 8 mice (24 h)	Scheirer-Ray-Hare test with two-tailed Mann-Whitney U post hoc	Time: χ^2 (1) = 5.1, p = .024<br>Treatment: χ^2 (1) = 3.3 p = .071	

		tests, Bonferroni-Holm correction	Interaction: χ^2 (1) = 1.9, *p* = .167<br><br>Vehicle-3 h vs DMG-3 h: *p* = .019<br>Vehicle-24h vs DMG-24h: *p* = .673<br>Vehicle-3h vs Vehicle-24h: *p* = .297<br>DMG-3h vs DMG-24h: *p* = .019	
9a	3 mice/group	Descriptive only		
9c	10 mice/group	Two-tailed unpaired t test	*T* (18) = 2.4, *p* = .029	
9d	**DMG:** 24 mice (Control) and 26 mice (DMG)<br>**HBL:** 14 mice (Control) and 13 mice (DMG)	**DMG:** Two-tailed Mann-Whitney U test<br>**HBL:** Two-tailed unpaired t test	**DMG:** *p* = .004<br>**HBL:** *T* (25) = 2.2, *p* = .037	Values excluded:<br>**DMG**: 3 outliers (Control) and 2 outliers (DMG)<br>**HBL**: 1 outlier (DMG)
9e	15 mice/group	Two-tailed unpaired t test	*T* (28) = 2.4, *p* = .023	
9f	**Control:** 15 mice<br>**STZ:** 17 mice	Two-tailed unpaired t test	*T* (30) = 2.8, *p* = .009	
10b	**Control:** 15 mice<br>**DMG:** 13 mice	Two-tailed unpaired t test	*T* (26) = 4.1, *p* = 4×10^{-4}	Values excluded: 2 outliers in DMG group
10d	**Control:** 15 mice<br>**STZ:** 14 mice	Two-tailed unpaired t test	*T* (27) = 2.1, *p* = .048	
10f	**Control:** 11 mice<br>**DMG:** 10 mice	Two-tailed Mann-Whitney U test	*p* = .030	
10g	**Control:** 14 mice<br>**DMG:** 15 mice	Two-tailed Mann-Whitney U test	*p* = .008	Value excluded: 1 outlier in Control group
10h	**Control:** 12 mice<br>**DMG:** 11 mice	Two-tailed Mann-Whitney U test	*p* = .032	
10i	Sum of areas of hippocampus:<br>**Control:** 19 sections/area<br>**DMG:** 20 sections/area<br><br>2 sections per mouse (15 mice)	Two-tailed Mann-Whitney U test	*p* = 6×10^{-4}	

Supplementary Table 6

Figure	Sample size (n)	Statistical test	Values	Comments
S1a	4 mice/group	**3-DG**, **Gx** and **MG**: Two-tailed unpaired t test<br>**DMG:** Two-tailed Mann-Whitney U test	**3-DG:** *T* (6) = 4.4, *p* = .005<br>**Gx:** *T* (6) = 2.0, *p* = .095<br>**MG:** *T* (6) = 0.8, *p* = .476<br>**DMG:** *p* = .029	
S1b	13 mice/group	Two-tailed Mann-Whitney U test	$p = 2 \times 10^{-7}$	
S1c	**DMG and pyruvate:** 12 mice (Control) and 11 mice (STZ)<br><br>**Lactate and lactate-pyruvate ratio**: 11 mice (Control) and 10 mice (STZ)	**DMG** and **lactate-pyruvate ratio:** Two-tailed unpaired t test<br>**Pyruvate** and **lactate**: Two-tailed Mann-Whitney U test	**DMG:** *T* (21) = 2.3, *p* = .030<br>**Pyruvate:** *p* = .023<br>**Lactate**: *p* = .350<br>**Lactate-pyruvate ratio:** *T* (19) = 2.9, *p* = .008	
S1d	**DMG, lactate and lactate-pyruvate ratio:** 13 mice (Control) and 12 mice (STZ)<br>**Pyruvate:** 12 mice/group	**DMG:** Two-tailed unpaired t test<br>**Pyruvate**, **lactate** and **lactate-pyruvate ratio:** Two-tailed Mann-Whitney U test	**DMG:** *T* (23) = 2.3, *p* = .028<br>**Pyruvate:** *p* = .160<br>**Lactate**: *p* = .728<br>**Lactate-pyruvate ratio:** *p* = .956	Value excluded: **Pyruvate:** 1 outlier (Control group)
S1f	**Body weight-time:** 12 mice/group<br>**Glucose**: 11 mice/group	**Body weight-time:** 2-way RM ANOVA<br>**Glucose:** Two-tailed Mann-Whitney U test	**Body weight-time:**<br>Time: *F* (2.2,47.5) = 457.6, $p < 1 \times 10^{-15}$<br>Genotype: *F* (1,22) = 75.8, $p = 1 \times 10^{-8}$<br>Interaction: *F* (6,132) = 109.0, $p < 1 \times 10^{-15}$<br><br>**Glucose:** $p = 3 \times 10^{-4}$	
S1g	**Standard diet**: 8 mice<br>**High fat diet:** 6 mice	**Body weight** and **DMG:** Two-tailed unpaired t test	**Body weight:** *T* (12) = 2.5, *p* = .028<br>**DMG:** *T* (12) = 0.5, *p* = .596	
S1h	**3-DG:** 98 patients (Diabetes)<br>**Gx:** 99 patients (Diabetes)<br>**MG:** 98 patients (Diabetes)<br>**DMG:** 99 patients (Diabetes)	Pearson correlation coefficient	**3-DG:** r = 0.38, $p = 9 \times 10^{-5}$<br>**Gx:** r = - 0.11, *p* = .291<br>**MG:** r = 0.03, *p* = .777<br>**DMG:** r = - 0.05, *p* = .643	
S1i	**Glucose:** 100 patients/group<br>**3-DG:** 100 patients (Control) and 99 patients (Diabetes)<br>**DMG:** 100 patients (Control) and 99 patients (Diabetes)	Receiver operating characteristic (ROC) curve	**Glucose:** AUC = 0.85 (0.793-0.903), *p* < .001, cut-off value = 6.6<br>**3-DG:** AUC = 0.79 (0.724-0.848), *p* < .001, cut-off value = 40.7	

	**MG:** 100 patients (Control) and 98 patients (Diabetes)<br>**Gx:** 100 patients (Control) and 99 patients (Diabetes)		**DMG:** AUC = 0.71 (0.640-0.782), *p* < .001, cut-off value = 1.87<br>**MG:** AUC = 0.65 (0.574-0.726), *p* < .001, cut-off value = 9.13<br>**Gx:** AUC = 0.63 (0.554-0.708), *p* = .001, cut-off value = 51	
S1j		Descriptive only		
S1k	10 mice/group	Scheirer-Ray-Hare test followed by two-tailed Mann-Whitney U post hoc tests, Bonferroni-Holm correction	**Food:**<br>Diet: χ^2 (1) = 13.4, *p* = 3×10^{-4}<br>Treatment: χ^2 (1) = 0.3, *p* = .572<br>Interaction: χ^2 (1) = 3.7, *p* = .055<br><br>Vehicle-chow vs. Vehicle-KD: *p* = .210<br>STZ -chow vs. STZ-KD: *p* = .006<br><br>**Water:**<br>Diet: χ^2 (1) = 5.7, *p* = .018<br>Treatment: χ^2 (1) = 8.6, *p* = .003<br>Interaction: χ^2 (1) = 6.3, *p* = .012<br><br>Vehicle-chow vs. Vehicle-KD: *p* = .912<br>STZ -chow vs. STZ-KD: *p* = 8×10^{-5}	
S2a	8 wells/group	Descriptive only/Two-tailed unpaired t test	*T* (14) = 0.02, *p* = .983	
S2b	8 wells/group	Descriptive only/Two-tailed unpaired t test	*T* (14) = 1.0, *p* = .326	
S2c-l		Descriptive only		
S2m	25 wells (Normoxia-Vehicle), 17 wells (Normoxia-Dichloroacetate) and 26 wells (Hypoxia-Vehicle)	One-way ANOVA followed by Dunnett´s post-hoc tests	*F* (2, 65) = 28.5, *p* = 1×10^{-9}<br>Normoxia-Vehicle vs. Normoxia-Dichloroacetate: *p* = .002<br>Normoxia-Vehicle vs. Hypoxia-Vehicle: *p* < 1×10^{-4}	Values excluded: 1 outlier in Normoxia-Vehicle group and 1 outlier in Normoxia-Dichloroacetate group
S2n	**$^{13}C_2$ DMG:** 20 wells (Normoxia-Vehicle), 14 wells (Normoxia-Dichloroacetate) and	One-way ANOVA followed by Dunnett´s post-hoc tests	**$^{13}C_2$ DMG:** *F* (2, 53) = 1.2, *p* = .297<br><br>**$^{13}C_4$ DMG:** *F* (2, 51) = 5.1, *p* = .010	Value excluded:<br>**$^{13}C_4$ DMG:** 1 outlier in

	22 wells (Hypoxia-Vehicle)<br>**$^{13}C_4$ DMG:** 19 wells (Normoxia-Vehicle), 14 wells (Normoxia-Dichloroacetate) and 21 wells (Hypoxia-Vehicle)		Normoxia-Vehicle vs. Normoxia-Dichloroacetate: p = .832<br>Normoxia-Vehicle vs. Hypoxia-Vehicle: p = .027	Hypoxia-Vehicle group
S3a	8 wells/group	**$^{13}C_2$ MG:** 2-way ANOVA<br>**$^{13}C_3$ MG:** Scheirer-Ray-Hare test	**$^{13}C_2$ MG:**<br>Transfection: F (1,28) = 4.1, p = .053<br>Oxygen: F (1,28) = 103.5, $p = 7 \times 10^{-11}$<br>Interaction: F (1,28) = 0.01, p = .912<br><br>**$^{13}C_3$ MG:**<br>Transfection: χ^2 (1) = 1.6, p = .200<br>Oxygen: χ^2 (1) = 23.3, $p = 1 \times 10^{-6}$<br>Interaction: χ^2 (1) = 0.02, p = .888	
S3b	3 mice/group	Descriptive only		
S3c	4 mice (*Ilvbl*$^{+/+}$) and 6 mice (*Ilvbl*$^{-/-}$)	Two-tailed unpaired t test	T (8) = 0.8, p = .448	
S3d	3 mice/group	Two-tailed unpaired t test	T (4) = 0.7, p = .533	
S3e	7 mice/group	Two-tailed unpaired t test	T (12) = 1.5, p = .170	
S3f	3 mice/group	Two-tailed Mann-Whitney U test	p = .700	
S3g	3 mice/group	Two-tailed unpaired t test	T (4) = 0.06, p = .954	
S3h	**Sham-Ilvbl$^{+/+}$:** 14 mice<br>**Sham-*Ilvbl*$^{-/-}$:** 17 mice<br>**MCAO-*Ilvbl*$^{+/+}$:** 12 mice<br>**MCAO-*Ilvbl*$^{-/-}$:** 10 mice	2-way RM ANOVA	**MCAO:**<br>Side: F (1,20) = 39.5, $p = 4 \times 10^{-6}$<br>Genotype: F (1,20) = 0.3, p = .592<br>Interaction: F (1,20) = 1.6, p = .219<br><br>**Sham:**<br>Side: F (1,29) = 0.4, p = .552<br>Genotype: F (1,29) < 0.01, p = .966<br>Interaction: F (1,29) = 0.4, p = .509	
S3i	**Sham-*Ilvbl*$^{+/+}$:** 14 mice<br>**Sham-*Ilvbl*$^{-/-}$:** 17 mice<br>**MCAO-*Ilvbl*$^{+/+}$:** 12 mice<br>**MCAO-*Ilvbl*$^{-/-}$:** 10 mice	2-way RM ANOVA	**MCAO:**<br>Side: F (1,20) = 14.1, p = .001<br>Genotype: F (1,20) < 0.01, p = .937<br>Interaction: F (1,20) = 0.5, p = .472<br><br>**Sham:**	

			Side: *F* (1,29) = 4.0, *p* = .056<br>Genotype: *F* (1,29) = 0.5, *p* = .506<br>Interaction: *F* (1,29) = 2.8, *p* = .107	
S3j	**Sham-*Ilvbl*$^{+/+}$:** 11 mice<br>**Sham-*Ilvbl*$^{-/-}$:** 14 mice<br>**MCAO-*Ilvbl*$^{+/+}$:** 9 mice<br>**MCAO-*Ilvbl*$^{-/-}$:** 5 mice	**MG:** Scheirer-Ray-Hare test<br>**Gx:** 2-way ANOVA	**MG:**<br>MCAO: χ^2 (1) = 5.5, *p* = .020<br>Genotype: χ^2 (1) = 0.3, *p* = .560<br>Interaction: χ^2 (1) = 0.8, *p* = .365<br><br>**Gx:**<br>MCAO: *F* (1,35) = 3.2, *p* = .081<br>Genotype: *F* (1,35) = 0.1, *p* = .779<br>Interaction: *F* (1,35) = 0.4, *p* = .542	
S3k	*Ilvbl*$^{+/+}$: 10 mice<br>*Ilvbl*$^{-/-}$: 11 mice	Two-tailed unpaired t test	**Infarct size:** *T* (19) = 0.05, *p* = .960<br>**Occludin:** *T* (19) = 0.04, *p* = .970	
S3l	*Ilvbl*$^{+/+}$: 18 mice<br>*Ilvbl*$^{-/-}$: 14 mice	2-way RM ANOVA followed by Sidak´s post-hoc tests (*p*-values adjusted for multiple comparisons)	Time: *F* (1,30) = 97.3, *p* = 6 × 10^{-11}<br>Genotype: *F* (1,30) = 0.2, *p* = .688;<br>Interaction: *F* (1,30) < 0.01, *p* = .933<br><br>Baseline-*Ilvbl*$^{+/+}$ vs 12 weeks-*Ilvbl*$^{+/+}$: *p* = 4 × 10^{-8}<br>Baseline - *Ilvbl*$^{-/-}$ vs 12 weeks -*Ilvbl*$^{-/-}$: *p* = 7 × 10^{-7}	
S3m	**3-DG and MG:** 16 mice (*Ilvbl*$^{+/+}$) and 10 mice (*Ilvbl*$^{-/-}$)<br>**Gx:** 16 mice (*Ilvbl*$^{+/+}$) and 9 mice (*Ilvbl*$^{-/-}$)	2-way RM ANOVA	**3-DG:**<br>Time: *F* (1,24) = 89.7, *p* = 1 × 10^{-9}<br>Genotype: *F* (1,24) = 0.1, *p* = .759<br>Interaction: *F* (1,24) = 0.6, *p* = .455<br><br>**Gx:**<br>Time: *F* (1,23) = 48.4, *p* = 4 × 10^{-7}<br>Genotype: *F* (1,23) = 1.6, *p* = .223<br>Interaction: *F* (1,23) = 1.3, *p* = .265<br><br>**MG:**<br>Time: *F* (1,24) = 1.1, *p* = .302<br>Genotype: *F* (1,24) = 0.1, *p* = .779<br>Interaction: *F* (1,24) = 1.5, *p* = .240	Values excluded:<br>**Gx:** 1 outlier at 12 weeks (value was also excluded at baseline)

S3n	*Ilvbl*$^{+/+}$: 18 mice<br>*Ilvbl*$^{-/-}$: 12 mice	Two-tailed unpaired t test	**Gx:** *T* (28) = 1.7, *p* = .100<br>**MG**: *T* (28) = 0.569, *p* = .574	
S4a	**Plasma**<br>**10 min:** 5 mice/group<br>**180 min:** 9 mice (PBS) and 11 mice (DMG)<br>**1440 min:** 9 mice (PBS) and 10 mice (DMG)<br><br>**Brain**<br>**10 min:** 5 mice/group<br>**180 min:** 5 mice (PBS) and 4 mice (DMG)<br>**1440 min:** 5 mice/group	Two-tailed Mann-Whitney U test/Descriptive only	**Plasma:** *p* = .008<br>**Brain:** *p* = .008	Values excluded:<br>**Plasma -180 min:** 2 outliers in PBS group<br>**Plasma – 1440 min:** 1 outlier in PBS group and 1 outlier in DMG group
S4b	24 wells (Control), 11 wells (5 µM DMG) and 12 wells (20 µM DMG, 50 µM DMG, 100 µM DMG and 200 µM DMG)	One-way ANOVA followed by Dunnett´s post-hoc tests (*p*-values adjusted for multiple comparisons)	F (5,77) = 83.6, $p < 1 \times 10^{-15}$<br><br>0 µM vs 5 µM DMG: *p* = .537<br>0 µM vs 20 µM DMG: *p* = .022<br>0 µM vs 50 µM DMG: $p < 1 \times 10^{-4}$<br>0 µM vs 100 µM DMG: $p < 1 \times 10^{-4}$<br>0 µM vs 200 µM DMG: $p < 1 \times 10^{-4}$	
S4c	6 wells (0 µM DMG, 5 µM DMG, 50 µM DMG, 500 µM DMG, 5 µM MG, 50 µM MG and 500 µM MG), 3 wells (10 µM H_2O_2) and 2 wells (no cells)	Kruskal-Wallis followed by Dunn´s post hoc tests (*p*-values adjusted for multiple comparisons)	χ^2 (8) = 39.1, $p = 5 \times 10^{-6}$<br><br>0 µM vs 5 µM DMG, *p* > .999<br>0 µM vs 50 µM DMG, *p* = .036<br>0 µM vs 500 µM DMG, $p = 2 \times 10^{-4}$<br>0 µM vs 5 µM MG, *p* > .999<br>0 µM vs 50 µM MG, *p* > .999<br>0 µM vs 500 µM MG, *p* = .064<br>0 µM vs 10 µM H_2O_2, *p* = .014<br>0 µM vs no cells, *p* > .999	
S4d	6 wells (0 µM DMG, 5 µM DMG, 50 µM DMG, 500 µM DMG, 5 µM MG, 50 µM MG and 500 µM MG), 3 wells (10 µM H_2O_2) and 2 wells (no cells)	Kruskal-Wallis with Dunn´s post hoc tests (*p*-values adjusted for multiple comparisons)	χ^2 (8) = 42.9, $p = 9 \times 10^{-7}$<br><br>0 µM vs 5 µM DMG, *p* > .999<br>0 µM vs 50 µM DMG, *p* = .053<br>0 µM vs 500 µM DMG, $p = 1 \times 10^{-5}$<br>0 µM vs 5 µM MG, *p* > .999	

			0 µM vs 50 µM MG, *p* = .216<br>0 µM vs 500 µM MG, *p* = .002<br>0 µM vs 10 µM H_2O_2, *p* = .004<br>0 µM vs no cells, *p* > .999	
S4e	**Motor cortex:**<br>PBS-3h, PBS-24h and DMG-24h: 18 samples/group<br>DMG-3h: 17 samples<br>**Somatosensory cortex:**<br>PBS-3h and PBS-24h: 18 samples/group<br>DMG-3h and DMG-24h: 17 samples/group<br><br>3 sections per mouse (6 mice)	**Motor cortex:**<br>2-way ANOVA followed by targeted Sidak´s post-hoc tests (*p*-values adjusted for multiple comparisons)<br>**Somatosensory cortex:**<br>Scheirer-Ray-Hare test followed by two-tailed Mann-Whitney U post hoc tests, Bonferroni-Holm correction	**Motor cortex:**<br>DMG: F (1,67) = 6.8, *p* = .011<br>Time point: F (1,67) = 1.7, *p* = .200<br>Interaction: F (1,67) = 1.7, *p* = .200<br><br>PBS-3h vs DMG-3h: *p* = .593<br>PBS-24h vs DMG-24h: *p* = .014<br><br>**Somatosensory cortex:**<br>DMG: χ^2 (1) = 9.5, *p* = .002<br>Time point: χ^2 (1) = 0.5, *p* = .823<br>Interaction: χ^2 (1) = 0.03, *p* = .870<br><br>PBS-3h vs DMG-3h: *p* = .053<br>PBS-24h vs DMG-24h: *p* = .040	
S4f	***Ccl2*:** 7 wells (0 µM DMG), 8 wells (100 µM DMG) and 5 wells (500 µM DMG)<br><br>***Tnf***: 8 wells/group<br><br>***Il1b***: 8 wells/group<br><br>***Il1a*:** 12 wells (0 µM DMG), 10 wells (100 µM DMG) and 8 wells (500 µM DMG)	Kruskal-Wallis followed by Dunn´s post hoc tests (*p*-values adjusted for multiple comparisons)	***Ccl2*:**<br>χ^2 (2) = 9.2, *p* = .005<br><br>0 µM vs 100 µM DMG: *p* > .999<br>0 µM vs 500 µM DMG: *p* = .027<br><br>***Tnf*:**<br>χ^2 (2) = 15.4, $p = 5 \times 10^{-4}$<br><br>0 µM vs 100 µM DMG: *p* > .999<br>0 µM vs 500 µM DMG: *p* = .002<br><br>***Il1b*:**<br>χ^2 (2) = 13.3, *p* = .001<br><br>0 µM vs 100 µM DMG: *p* > .999<br>0 µM vs 500 µM DMG: *p* = .002<br><br>***Il1a*:**	Values excluded:<br>***Ccl2*:** 1 outlier in 0 µM DMG<br>***Il1a*:** 1 outlier in 100 µM and 3 outliers in 500 µM DMG

			χ^2 (2) = 15.7, *p* = 4 × 10^{-4}<br><br>0 µM vs 100 µM DMG: *p* > .999<br>0 µM vs 500 µM DMG: *p* = 3 × 10^{-4}	
S4g	***Retnla***: 8 wells/group<br>***Arg1***: 8 wells (0 µM DMG), 7 wells (100 µM DMG) and 6 wells (500 µM DMG)	Kruskal-Wallis followed by Dunn´s post hoc tests (*p*-values adjusted for multiple comparisons)	***Retnla***:<br>χ^2 (2) = 17.2, *p* = 2 × 10^{-4}<br><br>0 µM vs 100 µM DMG: *p* = .358<br>0 µM vs 500 µM DMG: *p* = 1 × 10^{-4}<br><br>***Arg1***:<br>χ^2 (2) = 12.8, *p* = 2 × 10^{-4}<br><br>0 µM vs 100 µM DMG: *p* = .906<br>0 µM vs 500 µM DMG: *p* = .001	Values excluded:<br>***Arg1***: 1 outlier in 100 µM DMG and 2 outliers in 500 µM DMG
S4h	**Dentate gyrus:** 10 mice/group<br>**All hippocampus:** Dentate gyrus + CA	**Dentate gyrus:** Two-tailed unpaired t test<br>**All hippocampus:** Two-tailed Mann-Whitney U test	**Dentate gyrus:** *T* (18) = 1.0, *p* = .308<br>**All hippocampus:** *p* = .021	
S4i	**Motor cortex, Somatosensory cortex and Visual cortex:** 10 mice/group<br>**All-Cortex:** 30 sections/group	**Motor cortex, Somatosensory cortex and All-Cortex:** Two-tailed unpaired t test<br>**Visual cortex:** Two-tailed Mann-Whitney U test	**Motor cortex:** *T* (18) = 1.6, *p* = .118<br>**Somatosensory cortex:** *T* (18) = 0.3, *p* = .807<br>**Visual cortex:** *p* = .123<br>**All-Cortex:** *T* (58) = 2.3, *p* = .025	
S4j	4 mice/group<br><br>4 wells per mouse	One-way ANOVA followed by Dunnett´s post-hoc tests (*p*-values adjusted for multiple comparisons)	**3h:** *F* (2,45) = 18.5, *p* = 1 × 10^{-6}<br>Ctrl vs MG: *p* = .134;<br>Ctrl vs DMG: *p* = 3 × 10^{-4}<br><br>**24h:** *F* (2,45) = 2.0, *p* = .151	
S5b	**Control:** 27 mice<br>**DMG:** 28 mice	Two-tailed Mann-Whitney U test	*p* = .587	
S5c	**Control:** 13 mice<br>**DMG:** 14 mice	Two-tailed Mann-Whitney U test	*p* = .350	
S5d	**Control**: 15 mice<br>**STZ**: 17 mice<br><br>**DMG:** 15 mice/group	Mixed-effects analysis (REML)	**STZ:**<br>Time: *F* (1.7,46.5) = 40.0, *p* = 4 × 10^{-10}<br>Group: *F* (1,30) = 3.36, *p* = .077<br>Interaction: *F* (2,54) = 0.88, *p* = .420<br><br>**DMG:**	

			Time: *F* (1.9,53.3) = 34.5, *p* = 4 × 10^{-10}<br>Group: *F* (1,28) = 0.20, *p* = .656<br>Interaction: *F* (2,55) = 0.06, *p* = .945	
S5e	**Control**: 15 mice<br>**STZ**: 17 mice<br><br>**DMG:** 15 mice/group	Mixed-effects analysis (REML) followed by Sidak´s post hoc tests (*p*-values adjusted for multiple comparisons)	**STZ:**<br>Time: *F* (1.5,40.7) = 60.1, *p* = 2 × 10^{-11}<br>Group: *F* (1,30) = 11.9, *p* = .002<br>Interaction: *F* (2,54) = 0.6, *p* = .560<br><br>Control vs STZ – OF1: *p* = .035<br>Control vs STZ – OF2: *p* = .002<br>Control vs STZ – OF2: *p* = .108<br><br>**DMG:**<br>Time: *F* (1.9, 52.3) = 60.1, *p* = 6 × 10^{-14}<br>Group: *F* (1,28) = 3.2, *p* = .082<br>Interaction: *F* (2,55) = 0.2, *p* = .819	
S5f, upper panel (DMG)	15 mice/group	**Distance, speed and total exploration:** Two-tailed unpaired t test<br>**Time in central zone:** Two-tailed Mann-Whitney U test	**Distance:** *T* (28) = 1.2, *p* = .237<br>**Speed:** *T* (28) = 1.2, *p* = .242<br>**Time in central zone:** *p* = .345<br>**Total exploration:** *T* (28) = 0.3, *p* = .754	
S5f, lower panel (STZ)	**Control**: 15 mice<br>**STZ**: 17 mice	**Distance, speed:** Two-tailed unpaired t test<br>**Time in central zone, total exploration:** Two-tailed Mann-Whitney U test	**Distance:** *T* (30) = 1.8, *p* = .076<br>**Speed:** *T* (30) = 1.9, *p* = .070<br>**Time in central zone:** *p* = .123<br>**Total exploration:** *p* = .0496	
S5g	**Control, STZ**: 19 mice<br><br>**DMG**: 20 mice	Kruskal-Wallis test	χ^2 (2) = 2.7, *p* = .265	
S5h	**Control:** 13 mice<br>**DMG:** 14 mice	Pearson correlation coefficient	**Control:** r = - 0.75; *p* = .003<br>**DMG:** r = - 0.73; *p* = .003	
S5i	14 mice	Pearson correlation coefficient	r = 0.54; *p* = .044	
S5j	**Control, DMG**: 20 mice<br>**STZ**: 19 mice	One-way ANOVA	F (2,56) = 0.3, *p* = .715	

S5k	**Day 5:** 20 mice (Control and DMG) and 18 mice (STZ)<br>**Day 16:** 16 mice (Control), 20 mice (DMG) and 18 mice (STZ)	Kruskal-Wallis test	**Day 5:** χ^2 (2) = 3.8, *p* = .150<br>**Day 16:** χ^2 (2) = 2.8, *p* = .250	
S5l	**Day 5:** 19 (Control) 20 mice (DMG) and 18 mice (STZ)<br>**Day 16:** 16 mice (Control), 20 mice (DMG) and 17 mice (STZ)	Chi-squared test	**Day 5:** *p* = .042<br>**Day 16:** *p* = .031	
S5m	**Control:** 19 sections<br>**DMG:** 20 sections<br><br>2 sections for mouse (15 mice)	Two-tailed Mann-Whitney U test	**Dentate gyrus:** *p* = .026<br>**CA1**: *p* = .006	
